# Attenuated Lactococcus lactis and Surface Bacteria as Tools for Conditioning the Microbiota and Driving the Ripening of Semisoft Caciotta Cheese

**DOI:** 10.1128/AEM.02165-19

**Published:** 2020-02-18

**Authors:** Maria Calasso, Fabio Minervini, Francesca De Filippis, Danilo Ercolini, Maria De Angelis, Marco Gobbetti

**Affiliations:** a Department of Soil, Plant and Food Sciences, University of Bari Aldo Moro, Bari, Italy; b Department of Agricultural Sciences, University Federico II of Napoli, Naples, Italy; c Task Force on Microbiome Studies, University Federico II of Napoli, Naples, Italy; d Faculty of Science and Technology, Free University of Bozen, Bolzano, Italy; Centers for Disease Control and Prevention

**Keywords:** caciotta cheese, attenuated, lactococci, surface bacteria

## Abstract

This study aimed at establishing the effects of attenuated starters and surface bacteria on various features of caciotta cheese. The cheese undergoes a ripening period during which the house microbiota contaminates the surface. Conventional cheese (the control cheese [CC]) is made using only primary starters. Primary starters and attenuated (i.e., unable to grow and synthesize lactic acid) Lactococcus lactis (Lc. lactis) subsp. *lactis* were used to produce caciotta cheese without (ATT cheese) or with an inoculum of surface bacteria: (i) Leuconostoc lactis (Le. lactis) (LL cheese), (ii) Vibrio casei (VC cheese), (iii) Staphylococcus equorum (SE cheese), (iv) Brochothrix thermosphacta (BX cheese), and (v) a mixture of all four (MIX cheese). Attenuated Lc. lactis increased microbial diversity during cheese ripening. At the core, attenuated starter mainly increased indigenous lactococci and Lactobacillus delbrueckii group bacteria. At the surface, the main effect was on Macrococcus caseolyticus. Autochthonous Le. lactis strains took advantage of the attenuated starter, becoming dominant. Adjunct Le. lactis positively affected Lactobacillus sakei group bacteria on the LL cheese surface. Adjunct V. casei, S. equorum, and B. thermosphacta did not become dominant. Surfaces of VC, SE, and BX cheeses mainly harbored Staphylococcus succinus. Peptidase activities were higher in cheeses made with attenuated starter than in CC, which had the lowest concentration of free amino acids. Based on the enzymatic activities of adjunct Le. lactis, LL and MIX cheeses exhibited the highest glutamate dehydrogenase, cystathionine-γ-lyase, and esterase activities. As shown by multivariate statistical analyses, LL and MIX cheeses showed the highest similarity for microbiological and biochemical features. LL and MIX cheeses received the highest scores for overall sensory acceptability.

**IMPORTANCE** This study provides in-depth knowledge of the effects of attenuated starters and surface bacterial strains on the microbiota and related metabolic activities during cheese ripening. The use of attenuated Lc. lactis strongly impacted the microbiota assembly of caciotta cheese. This led to improved biochemical and sensory features compared to conventional cheese. Among surface bacterial strains, Le. lactis played a key role in the metabolic activities involved in cheese ripening. This resulted in an improvement of the sensory quality of caciotta cheese. The use of attenuated lactic acid bacteria and the surface adjunct Le. lactis could be a useful biotechnology to improve the flavor formation of caciotta cheese.

## Abstract (precis)

This study provides in-depth knowledge of the effects of attenuated starters and surface bacterial strains on the microbiota and related metabolic activities during cheese ripening. The use of attenuated Lc. lactis strongly impacted the microbiota assembly of caciotta cheese. This led to improved biochemical and sensory features compared to conventional cheese. Among surface bacterial strains, Le. lactis played a key role in the metabolic activities involved in cheese ripening. This resulted in an improvement of the sensory quality of caciotta cheese. The use of attenuated lactic acid bacteria and the surface adjunct Le. lactis could be a useful biotechnology to improve the flavor formation of caciotta cheese.

## INTRODUCTION

A cascade of biochemical and microbiological events occurs during ripening, the most important event of cheese manufacturing. Coagulant, milk-endogenous enzymes, and, especially, metabolic activities of the cheese microbiota mediate most of the biochemical reactions occurring in cheese during ripening (1, 2). Primary and secondary starters, nonstarter lactic acid bacteria (NSLAB) (3), adjunct/attenuated cultures (4–7), and microbial populations coming from the milk and cheese manufacturing environment shape the cheese microbiota (8–11). Biotic and abiotic drivers affect the establishment, assembly, and metabolism of the cheese microbiota, which is involved in the development of cheese sensory and nutritional features. Indeed, unbalanced microbiota and related metabolic activities may increase the risk of cheese off flavors and odors (9, 12–14). Deep investigation of the microbial interactions during cheese ripening allows the manufacture of cheeses with improved and consistent quality, reducing costs and improving appreciation by consumers (13, 15). Importantly, some biotechnological adjuvants used to accelerate cheese ripening might have an impact on the cheese sensory features (2). Attenuated cultures are represented by lactic acid bacteria that are unable to grow or synthesize significant amounts of lactic acid but act as sources of enzymes. Attenuated *Lactococcus* and *Lactobacillus* species are being successfully used to accelerate ripening and to improve flavor in a controlled manner (4–7). In particular, Lactococcus lactis (Lc. lactis), in the form of attenuated cells or cell extracts rich in enzymes, has received considerable interest, as it is the most used starter worldwide (5, 7). However, interactions among attenuated *Lactococcus* cells/enzymes and cheese microbiota during ripening need to be thoroughly investigated to avoid the growth of undesired indigenous microbes and to reduce variability in cheese properties (13, 16, 17).

The surfaces of all cheese varieties are colonized by a complex surface microbiota, which is equipped with suitable enzymatic activity and presents a barrier against pathogenic and spoiling microbes (15, 18). Mainly because of different ecosystem conditions (e.g., redox potential) and origins (inoculation or house microbiota), the cheese surface microbiota largely differs from the core microbiota (12, 19–21).

Manufactured in almost every Italian region, caciotta cheese is one of the most widespread traditional Italian varieties and is often used as a model system. The manufacture of caciotta cheese produces approximately 23,000 tonnes per year (https://www.assolatte.it). The cheese is mainly manufactured from pasteurized cow’s milk (sometimes in a mixture with ewe’s milk) inoculated with thermophilic starters (e.g., Streptococcus thermophilus and Lactobacillus delbrueckii subsp. *lactis*). Once salted, caciotta cheese undergoes ripening for approximately 15 days (fresh variety) or up to 2 months (aged variety; dry matter, approximately 60%). During ripening, the cheese surface is washed with brine. Caciotta cheese has a cylindrical shape, is 4 to 8 cm high and 8 to 16 cm in diameter, and weighs 0.8 to 2.0 kg. The surface of the aged variety is thin and yellow (22). The quality of the aged variety may occasionally not be acceptable because of blowing and off flavors caused by undesired fermentation and excessive concentrations of bitter oligopeptides, respectively. The use of attenuated adjunct cultures for caciotta cheese manufacture allowed control of ripening, positively influencing cheese flavor (6). Previously, the surface microbial community of caciotta cheese made using the primary starters S. thermophilus and L. delbrueckii subsp. *lactis* underwent investigation (21). Representatives from this community and from dairy plants (e.g., the ripening room) mainly belonged to *Leuconostoc* spp., *Vibrio* spp., *Brochothrix* spp., and Staphylococcus equorum. Surface contamination of the cheeses with appropriate mock microbial communities could be useful to elucidate the role of the surface microbiota during ripening of caciotta cheese.

This study aimed at establishing the effect of the inoculation of attenuated starters and surface bacterial strains on the microbiological, biochemical, and sensory features of caciotta cheese in comparison with conventionally manufactured caciotta cheese produced with pasteurized cow’s milk using S. thermophilus and L. delbrueckii subsp. *lactis* and brine salting (21). Experimental cheese variants were manufactured by adding attenuated cells of Lc. lactis CC01, with or without a surface inoculum of Leuconostoc lactis (Le. lactis), Vibrio casei, Brochothrix thermosphacta, and S. equorum, chosen as common contaminants of the dairy plant. An integrated biochemical and microbiological approach enabled the cheese characterization.

## RESULTS

### Kinetics of pH and water activity during cheese ripening.

Preliminary analyses showed that Le. lactis MC11, V. casei DSM22364, S. equorum DSM15097, and B. thermosphacta MC25 grew in a model system mimicking cheese and showed remarkable activities of enzymes involved in proteolysis and amino acid catabolism (see Table S1 in the supplemental material). Conventional caciotta cheese, made using pasteurized cow’s milk and inoculated with primary starters (S. thermophilus and L. delbrueckii subsp. *lactis*), was the control cheese (CC) (21). The experimental cheese variants used attenuated cells of Lc. lactis subsp. *lactis* CC01 (ATT cheese) and ATT and single surface inoculations (2 log CFU/cm^2^) of Le. lactis MC11 (LL cheese), V. casei DSM22364 (VC cheese), S. equorum DSM15097 (SE cheese), and B. thermosphacta MC25 (BX cheese) or a mixture of all four surface strains (MIX cheese). One day after manufacture, the moisture, fat, protein, and salt contents of CC were 48.1% ± 1.14%, 24.4% ± 0.97%, 21.5% ± 1.07%, and 0.47% ± 0.09%, respectively. The fat and protein levels trended inversely to the moisture content. After 30 days, the cheeses had average percentages of 25.8% ± 0.94% and 30.6% ± 0.27% for fat and protein, respectively. No significant differences (*P* ≥ 0.05) were observed for fat, protein, or salt among the cheese variants (data not shown).

**FIG 1:**
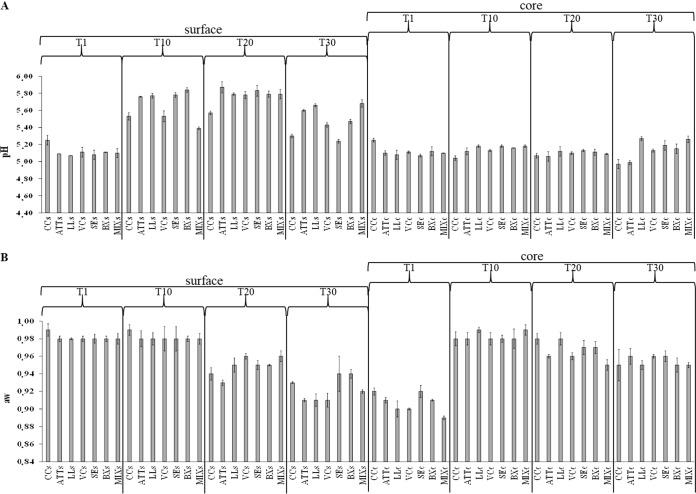
Evolution of pH (A) and *a_w_* (B) at 1 (T1), 10 (T10), 20 (T20), and 30 (T30) days of ripening at surface (s) and core (c) levels of CC and ATT, LL, VC, SE, BX, and MIX cheeses. The values represent the average (±standard deviation [SD]) of the results of analyses performed on three samples from as many cheese-making experiments.

The pH and water activity (*a_w_*) were recorded during cheese ripening (Fig. 1). One day after manufacture, the addition of attenuated cells increased (*P < *0.05) the acidification of the curd compared to CC. The pH values ranged from ca. 5.25 (surface and core of CC; *P > *0.05) to ca. 5.10 (surface and core of ATT cheese; *P > *0.05) (Fig. 1A). All the other cheese variants showed pH values similar (*P > *0.05) to those of ATT cheese. The kinetics of pH during ripening varied between cheese surface and core. After 20 days of ripening, the pH values of the cores did not differ between CC and ATT cheese samples. Surface-inoculated cheeses showed slightly higher pH values than ATT cheese. This trend persisted after 30 days of ripening, especially for LL and MIX cheese cores.

At the surface level, the pH value after 20 days was higher (*P < *0.05) in ATT cheese (5.8 ± 0.01) than in CC (5.6 ± 0.09) (Fig. 1A). Compared to ATT cheese, all the other cheese surfaces showed similar (*P > *0.05) pH values. These values further decreased at day 30. The ATT cheese surface had higher pH than the CC surface (5.6 ± 0.01 versus 5.3 ± 0.02; *P* = 0.045). SE cheese showed the lowest pH value, while LL and MIX cheeses showed the highest. The pH values of VC and BX cheese surfaces were lower than that of ATT cheese.

One day after manufacture, *a_w_* values, determined at both surface and core, did not differ (*P* > 0.05) among the cheeses (Fig. 1B). As expected, the *a_w_* values of the cheeses decreased during ripening. At core level, CC and ATT cheese showed the same *a_w_* values at 10 and 30 days. Compared to ATT cheese, *a_w_* values were higher in LL (10 and 20 days) and MIX (10 days) cheese core samples. As expected, at 20 and 30 days, surface samples showed lower *a_w_* values than core samples, probably also due to washing cheese surfaces with brine during ripening. The ATT cheese surface had lower *a_w_* values than CC during ripening.

### Cultivable microbiota.

**FIG 2:**
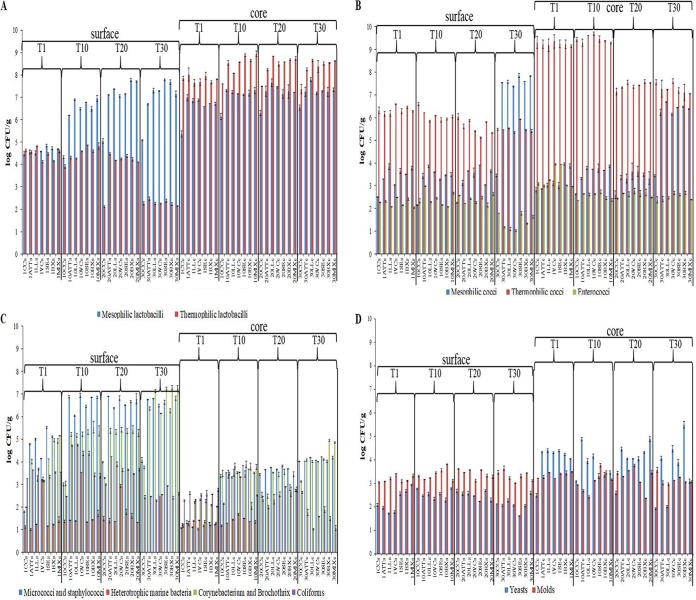
(A) Cell numbers (log CFU per gram) of presumptive mesophilic and thermophilic lactobacilli at surface (s) and core (c) levels of CC and ATT, LL, VC, SE, BX, and MIX cheeses after 1 (T1), 10 (T10), 20 (T20), or 30 (T30) days of ripening. The values represent the averages (±SD) of the results of analyses performed on three samples from as many cheese-making experiments. (B) Cell numbers (log CFU per gram) of presumptive mesophilic and thermophilic cocci and enterococci at surface and core levels of CC and ATT, LL, VC, SE, BX, and MIX cheeses after 1, 10, 20, or 30 days of ripening. The values represent the averages (±SD) of the results of analyses performed on three samples from as many cheese-making experiments. (C) Cell numbers (log CFU per gram) of presumptive micrococci and staphylococci, heterotrophic marine bacteria, *Corynebacterium* and *Brochothrix* isolates, and coliforms at surface and core levels of CC and ATT, LL, VC, SE, BX, and MIX cheeses after 1, 10, 20, or 30 days of ripening. The values represent the averages (±SD) of the results of analyses performed on three samples from as many cheese-making experiments. (D) Cell numbers (log CFU per gram) of presumptive yeasts and molds at surface and core levels of CC and ATT, LL, VC, SE, BX, and MIX cheeses after 1, 10, 20, or 30 days of ripening. The values represent the averages (±SD) of the results of analyses performed on three samples from as many cheese-making experiments.

Ten selective media were used to estimate cultivable bacteria, yeasts, and molds. Figure 2 shows the comparative microbial counts between surface and core. At day 1, cheese cores had a higher cell density of presumptive mesophilic and thermophilic lactobacilli than cheese surfaces (Fig. 2A). The ATT cheese core showed a higher number of mesophilic lactobacilli than CC at 1 day after manufacture. The cell density of mesophilic lactobacilli did not differ between ATT and surface-inoculated cheeses. Mesophilic lactobacilli increased during ripening, with the lowest value for CC. Overall, the surfaces of inoculated cheeses harbored a higher cell density of mesophilic lactobacilli than that of ATT cheese.

The presence of cultivable thermophilic lactobacilli in the cores of all the cheeses (except CC) increased during cheese ripening. Cheese surfaces showed an opposite trend. The ATT cheese core and surface showed higher numbers of cultivable thermophilic lactobacilli than those of CC after 20 days of ripening. After 20 days of ripening, the cores of surface-inoculated cheeses had contents of thermophilic lactobacilli higher than that found in ATT cheese.

At day 1, CC had the lowest presence of presumptive mesophilic cocci (Fig. 2B). This microbial group increased throughout cheese ripening. Except for CC, cheese surfaces at the end of ripening had higher levels of mesophilic lactococci than cheese cores. Compared to ATT cheese, all the other surface-inoculated cheeses showed similar (*P > *0.05) cell numbers of mesophilic lactococci.

Presumptive thermophilic cocci were found at higher numbers in cheese cores than on the surface (Fig. 2B). After 20 days of ripening, the microbial group decreased, especially in the cheese cores. With few exceptions, presumptive enterococci were always present at low cell density (<3 log CFU/g) (Fig. 2B).

Numbers of bacteria cultivable on marine agar were below 2.0 log CFU/g until 10 (cheese core) and 20 (cheese surface) days of cheese ripening (Fig. 2C). As expected, the only exception was found on the surface of the cheese inoculated with V. casei (VC cheese). Presumptive micrococci and staphylococci and bacteria cultivable on *Corynebacterium* agar were found at higher numbers on cheese surfaces than in cheese cores (Fig. 2C). The cheese with attenuated Lc. lactis cells added (ATT cheese) harbored a higher number of micrococci and staphylococci than CC, which increased during ripening and showed the highest level on the cheese surface. Presumptive *Corynebacterium* spp. and *Brochothrix* spp. increased, especially in all the cheeses with attenuated Lc. lactis cells added, reaching the highest numbers (ca. 7.0 to 7.5 log CFU/g) on the surfaces of LL, SE, BX, and MIX cheeses after 30 days of ripening. At day 1, presumptive coliforms ranged from ca. 2.5 (cheese core) to 3.5 (cheese surface) log CFU/g (Fig. 2C). Overall, coliforms decreased after 30 days of ripening, reaching the lowest values in cheese cores. Molds were variously distributed on cheese surfaces, ranging from ca. 2.5 to 3.8 log CFU/g (Fig. 2D). The highest yeast cell density was found in cores. Yeasts decreased during ripening of CC, whereas they were harbored in the highest numbers in the core of BX cheese at 30 days.

### Bacterial microbiome.

The bacterial community was monitored through high-throughput sequencing of the amplified V3-V4 region of the 16S rRNA gene. ATT cheese showed higher microbial diversity than CC (number of operational taxonomic units [OTUs], Chao1 richness, and Shannon index) (see Fig. S1 in the supplemental material). Compared with ATT cheese, all the other surface-inoculated cheeses showed similar (*P > *0.05) values of alpha diversity. Figure S2 in the supplemental material shows the beta diversity of the cheese bacterial community. Principal-coordinate analysis (PCoA) showed a clear differentiation of CC from the others. On the other hand, surface-inoculated cheeses were not distinguished from ATT cheese samples.

**FIG 3:**
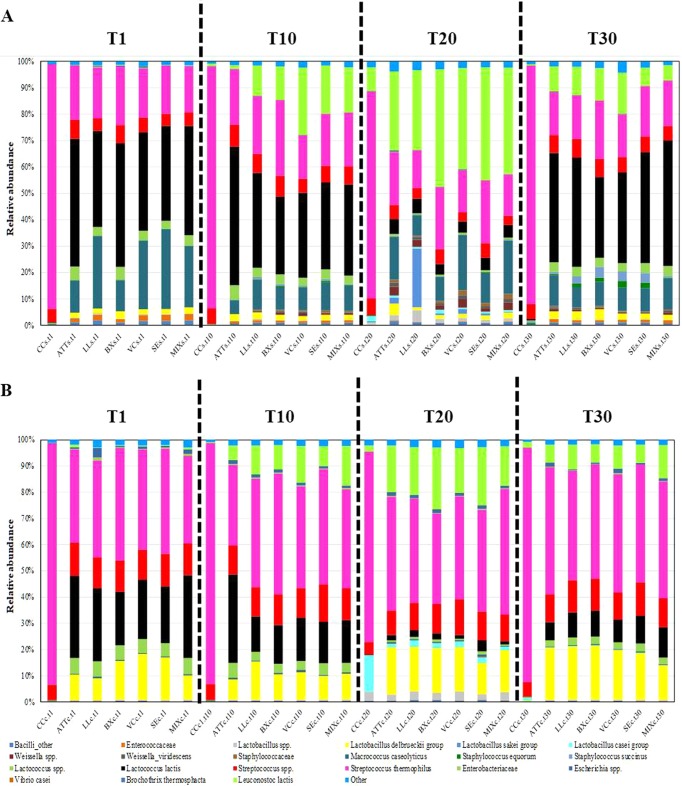
Relative abundances of total bacteria (genus/species level) found after 1 (T1), 10 (T10), 20 (T20), or 30 (T30) days of ripening at surface (A) and core (B) levels of CC and ATT, LL, VC, SE, BX, and MIX cheeses.

The taxa identified were included in six phyla (*Actinobacteria*, *Bacteroidetes*, *Firmicutes*, *Proteobacteria*, *Tenericutes*, and *Thermi*) and one candidate division (TM7). *Firmicutes* largely dominated the cheese core and surface, followed by *Proteobacteria*. The cheese core had a higher abundance of *Proteobacteria* than the cheese surface (reaching 5.21% in an LL cheese sample at 1 day of ripening). Within *Firmicutes*, *Streptococcaceae*, *Leuconostocaceae*, *Lactobacillaceae*, *Staphylococcaceae*, and *Enterococcaceae* were the dominant families (see Fig. S3 in the supplemental material). *Streptococcaceae* (mainly S. thermophilus) dominated the core and surface of CC (Fig. 3). The attenuated Lc. lactis cells seemed to affect the cheese microbiome. All cheeses made by the addition of attenuated cells of Lc. lactis showed lower relative abundance of S. thermophilus. At day 1, the relative abundance of Lc. lactis in the cheese cores varied from ca. 20.39 (BX cheese) to 31.52% (ATT cheese). Except for CC, the surfaces of all the cheese variants at day 1 had the highest presence of Lc. lactis, with percentages varying from ca. 35.80 (LL cheese) to 48.32% (ATT cheese). During ripening, Lc. lactis decreased until day 20, but then it significantly increased at day 30, especially on the cheese surface.

Compared to CC, the presence of attenuated Lc. lactis cells seemed to interfere with the increased relative abundance of the L. delbrueckii group, as revealed by linear discriminant analysis (LDA) of effect size (LEfSe) (see Fig. S4 to S11 in the supplemental material). The highest relative abundance of the L. casei group was in the CC core at 20 days of ripening (Fig. 3B; see Fig. S9). Compared to CC, the surfaces of all the other cheese variants subjected to the addition of the attenuated cells had a considerably higher relative abundance of Macrococcus caseolyticus and indigenous *Lactococcus* spp. (Fig. 3A; see Fig. S4, S6, S8, and S10). Also, the surfaces of LL, VC, SE, and MIX cheeses harbored M. caseolyticus at higher relative abundances than the ATT cheese surface. The lowest relative abundance of Le. lactis was in CC. At 10 days, indigenous and added Le. lactis bacteria increased in cheeses made using attenuated cells of Lc. lactis. Except for CC, Le. lactis increased throughout ripening, reaching the highest relative abundance on the cheese surface at 20 days (Fig. 3A; see Fig. S8). At that time, the surface of LL cheese harbored the highest level of Le. lactis, together with the Lactobacillus sakei group (Fig. 3A). S. equorum was mainly detectable on cheese surfaces, with the highest levels in SE and VC cheeses. At day 30, cheese surfaces, especially those of BX, VC, and SE cheeses, harbored Staphylococcus succinus. B. thermosphacta was found as a subdominant OTU mainly at the surface level of BX, VC, SE, and MIX cheeses.

By considering the species level (see Fig. S12 in the supplemental material) taxonomic assignments and significant correlations at a false-discovery rate (FDR) of <0.05, OTU cooccurrence was investigated. The most significant coexclusion patterns were identified for S. thermophilus and several OTUs belonging to *Firmicutes* (e.g., *Weissella*, *Macrococcus*, *Staphylococcus*, and *Lactococcus* species). The highest positive correlations were between Lc. lactis and M. caseolyticus, *Lactococcus* sp., *Enterococcaceae*, S. equorum, and S. succinus. Other significant positive correlations were found for *Leuconostoc*-L. sakei group and *Leuconostoc*-Weissella viridescens.

### Proteolysis and concentration of free amino acids.

Aminopeptidase type N (PepN), proline iminopeptidase (PepI), endopeptidase type O (PepO), glutamate dehydrogenase (GDH), cystathionine-γ-lyase (CGL), and esterase activities from water-soluble extracts of the cheeses during ripening were assessed using synthetic substrates (see Fig. S13 in the supplemental material). With few exceptions, enzyme activities were highest on the cheese surfaces. As expected, PepN, PepI, and PepO activities were higher in the cheeses made with attenuated cells of Lc. lactis than in CC, especially at 20 days of ripening. PepO activity was highest in ATT, LL, and VC cheeses. According to the estimated cell density (Fig. 2B) and enzymatic activities (see Table S1) of Le. lactis MC11, LL and MIX cheeses exhibited the highest (*P < *0.05) levels of GDH, CGL, and esterase activities, especially after 30 days of ripening.

**FIG 4:**
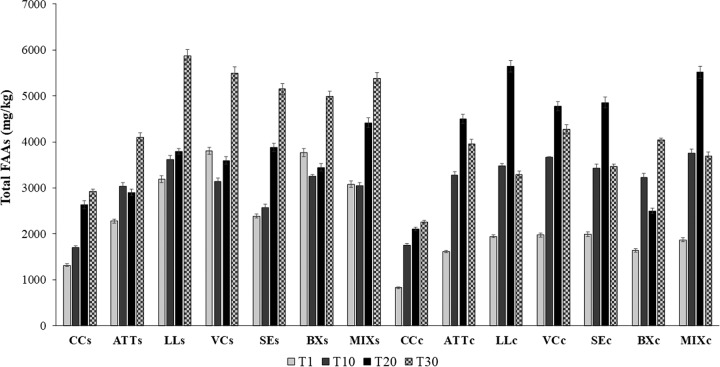
Total free amino acid concentrations found at surface (s) and core (c) levels of CC and ATT, LL, VC, SE, BX, and MIX cheeses after 1 (T1), 10 (T10), 20 (T20), or 30 (T30) days of ripening. The values represent the averages (±SD) of the results of analyses performed on three samples from as many cheese-making experiments.

**FIG 5:**
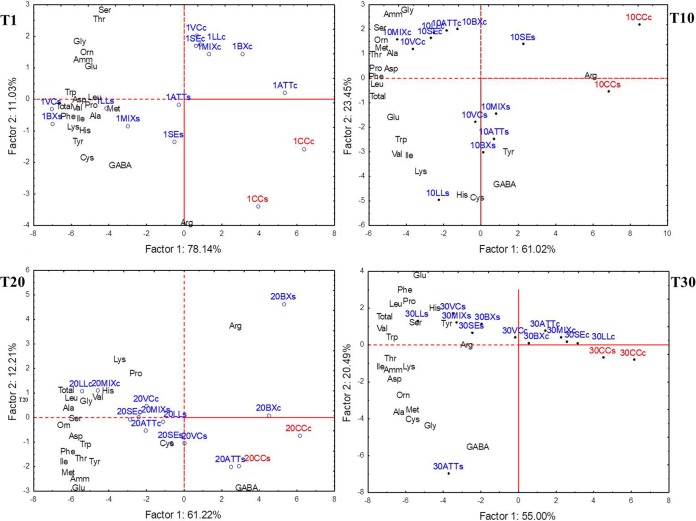
Scores and loading plots of the two PCA of the total and individual free amino acids and their main derivatives, which were found at the surface (s) and core (c) levels of CC (red) and ATT, LL, VC, SE, BX, and MIX cheeses (blue) after 1 (T1), 10 (T10), 20 (T20), or 30 (T30) days of ripening.

The concentration of peptides increased throughout ripening (see Fig. S14 in the supplemental material). CC contained a lower concentration of peptides than the other variants. The highest levels of peptides were found for ATT, LL, SE, and MIX cheeses. The concentration of free amino acids (FAAs) increased throughout ripening (Fig. 4). At day 1, the cheeses manufactured with attenuated cells or with attenuated cells and surface cultures exhibited concentrations of FAAs approximately two times higher than that found in CC. In particular, the surfaces of LL, VC, BX, and MIX cheeses had the highest levels of FAAs, while FAAs did not differ (*P* > 0.05) in the cores of ATT, LL, VC, SE, BX, and MIX cheeses. Overall, cheese cores had the highest concentrations of FAAs at day 20. The only exceptions were for CC and BX cheese. After 30 days of ripening, both the core and surface of CC had the lowest (*P* < 0.05) concentration of total FAAs (ca. 2,200 and 2,900 mg/kg, respectively). For the other cheese variants, FAAs ranged from approximately 4,100 mg/kg (ATT cheese surface) to 5,872 mg/kg (LL cheese surface) and from 3,287 mg/kg (LL cheese core) to 4,275 mg/kg (VC cheese core). The levels of several individual FAAs differed among cheeses (Fig. 5; see Table S2 in the supplemental material), which was mainly related to the addition of attenuated Lc. lactis cells and surface bacteria and time of ripening.

### Correlations between pH, water activity, cultivable microbiota, microbiome, and proteolysis of cheeses.

**FIG 6:**
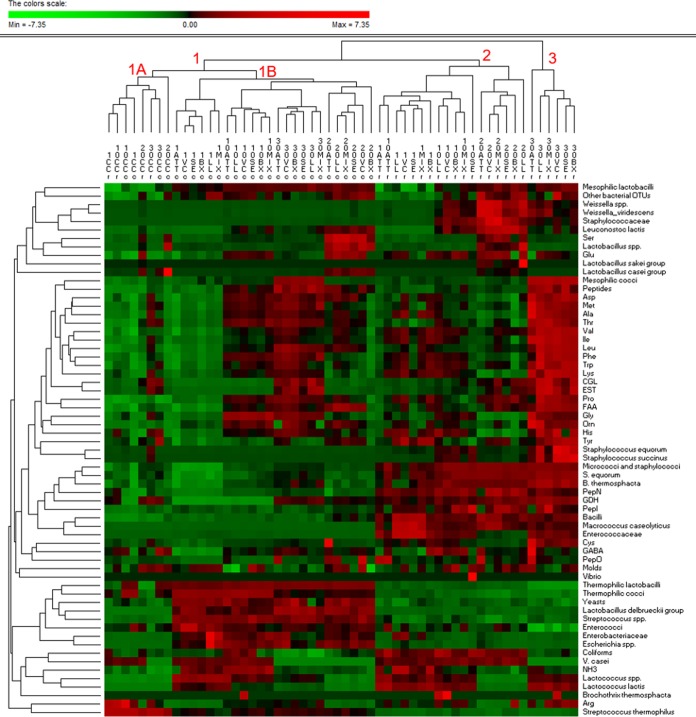
Permutation analysis based on the cultivable microbiota, dominant bacterial microbiome, enzymatic activities, and concentrations of peptides and individual and total free amino acids and their derivatives, which were found at surface (s) and core (c) levels of CC and ATT, LL, VC, SE, BX, and MIX cheeses after 1, 10, 20, or 30 days of ripening. Amino acids are indicated by three-letter codes; GABA, gamma aminobutyric acid; EST, esterase. Euclidean distance and McQuitty’s criterion (weighted pair group method with averages) were used for clustering. The colors correspond to normalized mean data levels from low (green) to high (red). The color scale, in terms of units of standard deviation, is shown at the top.

The results of analyses (cultivable microbiota, dominant bacterial microbiome, pH, *a_w_*, enzymatic activities, and concentrations of peptides and FAAs) performed for all the cheeses during the whole ripening period were used as entries for multivariate statistical analyses. Permutation analysis (Fig. 6) and principal-component analysis (PCA) (see Fig. S15 in the supplemental material) distinguished the surface and core of CC from those of the other variants. The outstanding dominance of S. thermophilus, together with the lowest values of some microbiological (e.g., mesophilic lactobacilli and cocci, micrococci and staphylococci, and relative abundances of the L. delbrueckii group and Le. lactis) and biochemical (e.g., enzymatic activities and FAAs) data, characterized CC (Fig. 6, cluster 1A). The cores of the other cheese variants grouped together because of the high cell density of thermophilic cocci and mainly lactobacilli and yeasts; low numbers of micrococci and staphylococci; and high PepN, PepI, and GDH activities (Fig. 6, cluster 1B). A further clustering of cheese cores agreed with the time of cheese ripening. The core of ATT cheese always differed from those of the other variants. At 20 and 30 days of ripening, the highest similarity was for cores of LL and MIX cheeses. Except for CC, all the other cheese surfaces were clearly distinguished from the cheese cores (Fig. 6, clusters 2 and 3). At day 30, cheese surfaces separated from the others previously sampled based on the high level of mesophilic cocci and staphylococci and lower numbers of coliform and V. casei bacteria, together with higher concentrations of almost all the FAAs, peptides, and enzyme activities (CGL and esterase). As shown for cheese cores, the surfaces of ATT cheeses showed distinct profiles. At the end of ripening, the highest similarity among cheese surfaces was between LL and MIX cheeses.

**FIG 7:**
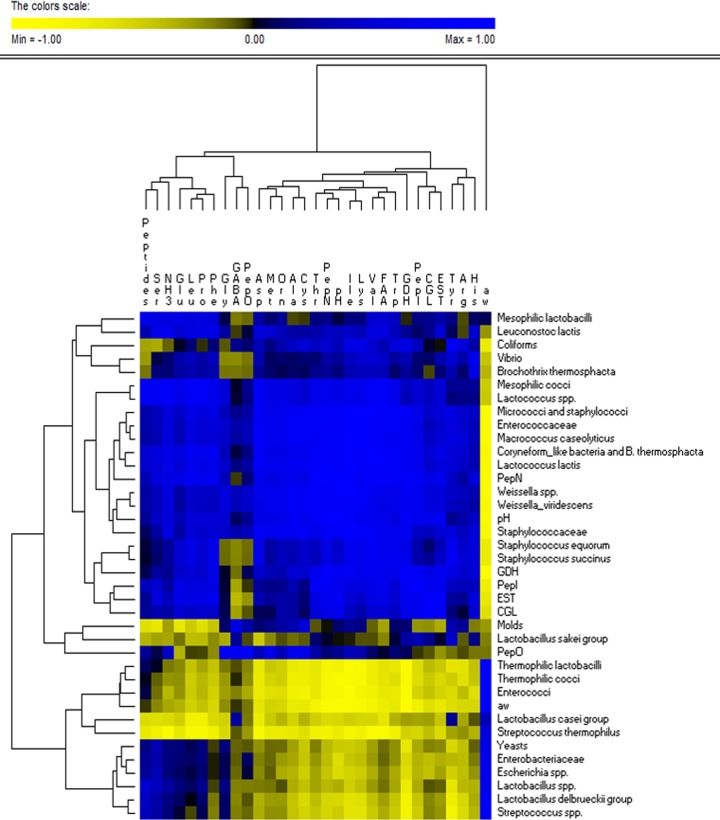
Correlations between cultivable microbiota, dominant bacterial microbiome, enzymatic activities, and concentrations of peptides and individual and total free amino acids and their derivatives that were found, after 30 days of ripening, at surface and core levels of CC and ATT, LL, VC, SE, BX, and MIX cheeses. Amino acids are indicated with three-letter codes. The color of the scale bar denotes the nature of correlation, with 1 indicating a perfectly positive correlation (blue) and −1 indicating a perfectly negative correlation (yellow). Euclidean distance and McQuitty’s criterion (weighted pair group method with averages) were used for clustering.

Considering the above-mentioned microbiological and biochemical results of the analyses performed on all the cheeses during ripening, some correlations between bacterial genera/species, cheese enzyme activities, and FAAs were found (Fig. 7). Mesophilic lactobacilli and lactococci, micrococci and staphylococci, and Le. lactis were positively correlated with enzyme activities (PepN, PepI, PepO, GDH, and CGL) and FAAs. Both enzymatic activities and the levels of individual FAAs were negatively correlated with the *a_w_* values. The *a_w_* value was positively correlated with most of the OTUs attributed to *Lactobacillus*, *Streptococcus*, *Escherichia*, and *Enterobacteriaceae*, whereas it was negatively correlated with *Vibrio*, B. thermosphacta, M. caseolyticus, *Staphylococcaceae*, and *Weissella*.

### Sensory analysis.

**FIG 8:**
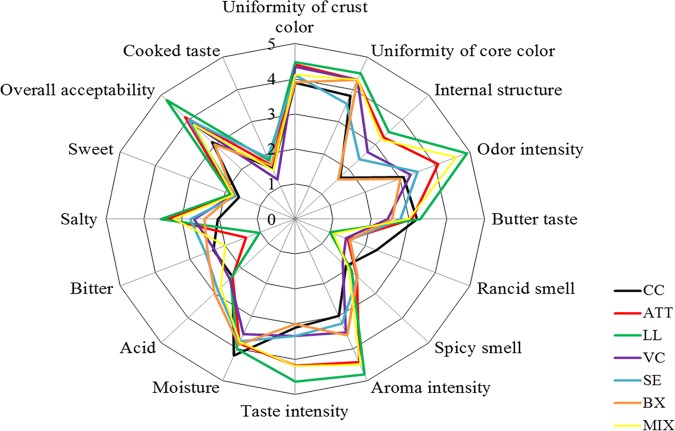
Sensory analysis performed after 30 days of ripening on CC and ATT, LL, VC, SE, BX, and MIX cheeses.

All the cheeses showed the typically desirable sensory characteristics of short-ripened caciotta cheese, namely, uniformity of color and structure, odor and taste recalling those of milk and butter, and absence of rancid and spicy smells, as well as of bitter taste (22). However, compared to CC, the use of attenuated Lc. lactis impacted the sensory features of caciotta cheese (Fig. 8). Cheeses manufactured with the addition of attenuated Lc. lactis cells and surface adjunct cultures displayed high scores for odor and aroma intensity and salty attributes. MIX and, especially, LL cheeses received higher scores for aroma and taste intensity and overall acceptability than ATT cheese. On the other hand, BX cheese received lower scores for uniformity of crust color, odor and aroma intensity, and overall acceptability than ATT cheese.

## DISCUSSION

This study used primary starters (S. thermophilus and L. delbrueckii subsp. *lactis*), attenuated Lc. lactis cells, and adjunct surface cultures (Le. lactis, V. casei, S. equorum, and B. thermosphacta) belonging to common cheese surface microbiota to make caciotta cheese. Cheese manufacturing was carried out at the same dairy plant, with the same biotic (house microbiota) and abiotic (e.g., milk composition, salt concentration, ripening temperature, and humidity) selection pressures, which may shape the microbial community (13, 14).

According to previous studies (4, 7, 13), attenuation rendered Lc. lactis CC01 cells unable to grow and to synthesize significant levels of lactic acid during cheese making. At the same time, the attenuated cells delivered enzymes (PepN, PepI, PepO, and GDH) that are mainly responsible for proteolysis and catabolism of FAAs. In general terms, ripening conditions (e.g., *a_w_*, NaCl concentration, and oxygen availability) and procedures adopted during ripening (e.g., brine salting and washing of cheese surfaces) shape the cheese microbiota and, in turn, the potential for enzymatic activities (8, 12). Differences between the cheese surface and core are common, especially for those varieties that do not undergo excessive surface drying during ripening (13, 14, 23). In this study, culture-dependent and -independent analyses allowed us to obtain an integrated overview of the bacterial dynamics at the surface and core during cheese ripening. Overall, the results of the two approaches overlapped, although a few differences appeared. One of the differences regarded *Brochothrix*, *Corynebacterium*, *Micrococcus*, and *Staphylococcus*, which were found at increasing cell numbers during ripening but whose relative abundances, determined through high-throughput 16S rRNA sequencing, were very low. Some drawbacks to the high-throughput-sequencing technique, such as preferential PCR amplification, different lysis efficiencies during extraction of DNA, and different numbers of copies of 16S rRNA operons among bacteria, could have caused such discrepancies (24). The addition of attenuated Lc. lactis cells was linked to increased microbial diversity and to quicker growth of autochthonous and/or starter lactic acid bacteria as early as day 1 of cheese manufacture. This may be attributed to lactococcal proteolysis and the consequent release of peptides and FAAs, which promoted the growth of cheese autochthonous bacteria. In the cheese core, the addition of attenuated Lc. lactis cells was related to an increased level of indigenous lactococci and the L. delbrueckii group. On the other hand, the main effect on the cheese surface was on M. caseolyticus. M. caseolyticus (family *Staphylococcaceae*) usually originates from animal organs, the farm environment, milk, and cheese (25–28). M. caseolyticus was previously found on the surfaces of Italian cheeses, such as taleggio, gorgonzola, casera, scimudin, and formaggio di fossa (29), and it is probably able to break down caseins, contributing to the formation of aroma precursors (30). M. caseolyticus was not detected during the manufacture of traditional caciotta cheese (21). On the other hand, in this study, it prevailed on the surfaces of all the cheese variants for up to 30 days. Significant positive correlations emerged between M. caseolyticus and other surface microbiome members, including Le. lactis and S. equorum. Compared to conventionally manufactured caciotta cheese (used as a control), the differences found in the cheeses made with attenuated cells were not attributable to the stochastic assembly of the microbiota across replicates because of their constant presence in all the replicate manufactures (14).

Variations in the assembly of the cheese microbiome were also evident when using the adjunct surface cultures of Le. lactis, V. casei, S. equorum, and B. thermosphacta, mimicking possible contamination through the environmental microbiota. Le. lactis and S. equorum are available as commercial cultures for cheese making to enhance the flavor and color of surface-ripened cheeses (31–33). The surface cultures used in this study showed the ability to grow under simulated cheese-ripening conditions and displayed some enzymatic activities involved in proteolysis, catabolism of FAAs, and lipolysis. To date, catalogues of Le. lactis, S. equorum, V. casei, and B. thermosphacta genomes have revealed the presence of some genes with high potential in cheese ripening, mainly for surface-ripened cheeses (18). Under the experimental conditions used in this study, S. equorum, V. casei, and B. thermosphacta did not show the capability to become dominant on the cheese surface, even if surface inoculated. This could be attributed to intrinsically higher fitness of the indigenous microbiota (e.g., *Lactococcus* spp. and S. succinus) contaminating the cheese-making plant, as well as to the poor adaptation of the inoculated bacteria. Indeed, unlike the other strains used in this study, which had been isolated from caciotta cheese, S. equorum and V. casei originated from the surfaces of different cheeses. Le. lactis inoculated on cheese surfaces seemed to take advantage of attenuated Lc. lactis cells, thus becoming a dominant component of the microbiome at the cheese core and, mainly, on the cheese surface. After 20 days of ripening, it reached the highest level of relative abundance on the cheese surface. Le. lactis was found as a dominant bacterial OTU in all the cheeses made with attenuated Lc. lactis cells, which suggested the prevalence of other autochthonous strains. The autochthonous strains did not show any prevalence in conventional caciotta cheese used as the control. Significant cooccurrence patterns were evident among Lc. lactis, Le. lactis, and the L. sakei group. A stable microbial community, mainly consisting of Lc. lactis, Leuconostoc mesenteroides, and Lactobacillus fuchuensis (belonging to the L. sakei group), was featured on the semihard Iranian Liqvan cheese (34). After 20 days of ripening, the L. sakei group was one of the dominant OTUs of the surface microbiome of only cheese made with the surface inoculation of Le. lactis. This led to the hypothesis of a strong interaction between the L. sakei group and Le. lactis MC11, rather than with autochthonous strains of the same species. Surface inoculation with cultures of S. equorum and V. casei resulted in a significant cooccurrence of Lc. lactis, *Lactococcus* sp., *Enterococcaceae*, and S. succinus. Surfaces of cheeses inoculated with cultures of B. thermosphacta, S. equorum, and V. casei mainly harbored S. succinus. Although very sensitive to biotic and abiotic factors (35), this species, together with other staphylococci, dominated in some European ripened cheese varieties (36). Although we found yeasts and molds at lower cell density than most bacterial groups, the fungal microbiota, through deacidification and production of vitamins and amino acids, may favor some Gram-positive, less acid-tolerant bacteria, including staphylococci and corynebacteria (15).

The microbiome assembly correlated well with several biochemical features (e.g., enzymatic activities and FAAs) of the cheeses. Previously, it was shown that during manufacture and ripening of Gouda cheese, the growth of L. mesenteroides relies on the proteolysis of Lc. lactis, which provides small peptides and essential FAAs (16). The liberation of FAAs increased when mesophilic lactic acid bacteria (e.g., Lc. lactis) combined with GDH-positive NSLAB (13). In this study, the diverse enzyme portfolios of different microbial communities resulted in distinct profiles for individual FAAs. The synthesis/liberation of FAAs constitutes a key factor affecting cheese flavor and the catabolism of FAAs, with the liberation of volatile organic compounds (2, 37).

As shown by sensory analysis, the addition of attenuated Lc. lactis CC01 cells improved cheese flavor. Cheese manufactured using attenuated cells and surface inoculation of B. thermosphacta was less acceptable than that made under conventional processing. Cheese made using attenuated cells and surface inoculation of Le. lactis or using a mixture of the four surface contaminants showed similar biochemical features and received the highest scores, including overall acceptability. It could be hypothesized that the high scores attributed to LL and MIX cheeses could be due to some positive metabolic interactions among *Leuconostoc*, *Corynebacterium*, *Brochothrix*, *Micrococcus*, and *Staphylococcus* cultures, which were found at the highest cell densities on the surfaces of LL and MIX cheeses. This result strengthens the importance of Le. lactis as an adjunct culture in cheese making (31, 38, 39) and confirms the contribution to cheese flavor and color by some surface bacterial genera (e.g., *Corynebacterium* spp.) (40).

This study highlighted the fact that the use of attenuated Lc. lactis cells and bacteria contaminating surfaces affects the microbial community assembly. The microbial assembly and function varied depending on space (surface/core) and time of ripening (1 to 30 days), affecting the enzymatic portfolio of cheeses. In turn, the specific dynamic of biochemical features drove the sensory profile of caciotta cheese. The use of attenuated lactic acid bacteria and surface adjunct Le. lactis could be a useful biotechnology to improve the flavor formation of caciotta cheese. However, since autochthonous microorganisms originating from milk or the dairy environment could vary across processing plants and seasons, appropriate experimentation should be designed in order to extend the results of this study to all cheeses. In addition, future studies in the field should include a culture-independent approach based on rRNA transcripts of taxonomically relevant genes, also extended to the fungal microbiome, to detect the metabolically active microbial population, including viable but noncultivable cells.

## MATERIALS AND METHODS

### Attenuated and surface adjunct cultures.

Lc. lactis subsp. *lactis* CC01, Le. lactis MC11, V. casei DSM22364 (biosafety level 1), S. equorum DSM15097, and B. thermosphacta MC25 (biosafety level 1) were used. Lc. lactis CC01, Le. lactis MC11, and B. thermosphacta MC25, from the Culture Collection of the Department of Soil and Food Sciences of Bari University, Bari, Italy, were previously isolated from caciotta cheese and identified by partial sequencing of the 16S rRNA. V. casei and S. equorum, isolated from milk products, were purchased from the Leibniz Institute DSMZ-German Collection of Microorganisms and Cell Cultures (Braunschweig, Germany). The propagation of all strains was at 30°C for 24 h, using different culture media. Specifically, Lc. lactis subsp *lactis* CC01 was grown on M17 broth (Oxoid, Basingstoke, United Kingdom); Le. lactis MC11 on de Man, Rogosa, and Sharpe (MRS) broth (Oxoid); V. casei DSM22364 on marine broth (Becton, Dickinson Italia, Milan, Italy) with shaking at 180 rpm; B. thermosphacta MC25 on *Corynebacterium* broth (casein peptone, 10.0 g/liter tryptic digest, 5.0 g/liter yeast extract, 5.0 g/liter glucose, 5.0 g/liter NaCl, pH 7.2 to 7.4); and S. equorum DSM15097 on Trypticase soy yeast extract (TSYE) broth (Merck KGaA, Darmstadt, Germany). Stock cultures were stored at −80°C, with 200 μl/ml glycerol as a cryoprotective agent.

To achieve attenuation, cells of Lc. lactis subsp. *lactis* CC01 (9.0 ± 0.35 log CFU/ml) grown overnight in M17 broth were harvested by centrifugation (10,000 × *g*; 10 min; 4°C); washed twice with sterile 50 mM potassium phosphate buffer, pH 7.0; and resuspended in distilled water at a density of ca. 11 log CFU/ml. The cell suspension was placed in an ice bath and subjected to sonication (Vibra-Cell sonicator; Sonic and Materials Inc., Danbury, CT, USA) with a microtip setting (sonic power, 375 W; output control, 5) for 15 min (3 cycles; 5 min/cycle) (6). As estimated by plate counting, sonication decreased cell survival to ca. 5.6 ± 0.1 log CFU/ml. After treatment, the preparation (containing free enzymes and unbroken cells) was freeze-dried, resuspended in sterile milk, and used to inoculate the cheese milk. The unbroken cells were unable to overacidify the cheese but represented an additional source of enzymes.

To prepare surface adjunct cultures, cells of Le. lactis, V. casei, B. thermosphacta, and S. equorum were harvested by centrifugation (10,000 × *g*; 10 min; 4°C); washed twice with sterile 50 mM potassium phosphate buffer, pH 7.0; and resuspended in water containing 0.85% NaCl. Inoculation of the cheese surface before ripening was at a density of ca. 2 log CFU/cm^2^. Bacterial suspensions were also combined (25% [vol/vol] each strain), and the four-strain cocktail was used (MIX). The level of inoculation was determined by plating each inoculum on the respective medium, namely, MRS agar for Le. lactis, marine agar for V. casei, *Corynebacterium* agar for B. thermosphacta, and TSYE agar for S. equorum. Enumeration was performed after 24 h of incubation at 30°C.

### Cheese manufacture and sampling.

Cheese making at an industrial plant (Ignalat srl, Noci, Bari, Italy) was carried out in triplicate on three consecutive days (three batches for each variant of cheese), using the conventional caciotta cheese protocol (21). The cow’s milk used had the following characteristics: lactose, 4.9%; protein, 3.3%; fat, 3.6%; pH 6.6. No significant (*P > *0.05) differences in compositional values emerged among the three batches of milk. The milk was pasteurized using a plate heat exchanger. Prior to and after use, the equipment was sterilized by circulation of water (at 85°C) for at least 30 min (41). Four hundred liters of milk was heat treated (71°C for 15 s). Total mesophilic aerobic microorganisms, found at levels of ca. 4.5 log CFU/ml in the raw milk (data not shown), were inactivated by pasteurization. After the heat treatment, the milk was instantaneously cooled to 37°C and inoculated with commercial primary starters: S. thermophilus and L. delbrueckii subsp. *lactis* (initial densities, approximately 7.5 ± 0.11 and 7.3 ± 0.14 log CFU/ml, respectively; Sacco, Cadorago; Como, Italy). Immediately after inoculation, the attenuated Lc. lactis culture (initial density, approximately 5.6 ± 0.1 log CFU/ml) was added to the milk tank, followed by gentle blending. This was the first variant of cheese, namely, the one with the attenuated strain added (ATT cheese). For CC, the addition of attenuated cells of Lc. lactis culture did not occur. After incubation for 30 min at 37°C, liquid calf rennet (35 ml/100 liters) was added, and coagulation took place within 30 min. After whey drainage and molding, the curds were stored for approximately 4 h at room temperature. Salting was done by immersing (5 h) the cheeses in brine (37% [wt/vol] NaCl). The salted ATT curds also underwent surface inoculation (2 log CFU/cm^2^) of (i) Le. lactis (LL cheese), (ii) V. casei (VC cheese), (iii) B. thermosphacta (BX cheese), (iv) S. equorum (SE cheese), or (v) a mixture of Le. lactis, V. casei, B. thermosphacta, and S. equorum (MIX cheese). These corresponded to the other five variants of caciotta cheese. There was no surface inoculation for CC cheese and the first variant of ATT cheese. The entire surfaces of LL, VC, BX, SE, and MIX cheeses were inoculated, using an L-shaped spreader, with 500 μl of bacterial cell suspension obtained, as described above, from liquid cultures incubated at 30°C for 24 h. The inoculated cheeses were kept at room temperature for 45 min to favor the attachment of the bacteria to the cheese surface (42). All the cheeses were left at room temperature for a further 12 h. The cheeses weighed approximately 1.5 kg. Ripening was at 9°C (relative humidity, ca. 73%) for 30 days. All cheese variants were repeatedly (3 or 4 times) turned and washed with a brine solution (20% [wt/vol] NaCl) during ripening. Specifically, washing of the cheese surface was performed using a cotton cloth (one for each cheese variant) soaked in brine (43). For each batch, cheese sampling was performed at 1, 10, 20, and 30 days of ripening. All the analyses considered both the cheese surface and core (ca. 100 g). The only two exceptions were for raw chemical composition and sensory analysis, which were determined on whole (surface and core) cheese samples. For the cheese surface, the rind was removed by scraping the entire surface of each cheese wheel with sterile scalpels to about 5-mm depth (18, 44). Cheese cores were obtained after removing the first 2 cm of the surface (23). All samples were transported to the laboratory under refrigerated conditions (∼4°C) and immediately subjected to compositional, microbiological, and biochemical analyses. An aliquot of each sample was frozen (−80°C) until culture-independent analysis was performed.

### Analyses of compositional and cultivable microbiota.

Three samples of CC (including rind and core) 1 day after manufacture and three samples of all the cheeses (including rind and core) after 30 days of ripening were analyzed for protein (macro-Kjeldahl) (45), fat (Gerber method) (46), moisture (oven drying at 102°C) (47), and salt (48). The pH values and water activity (*a_w_*) were determined on the rinds (surfaces) and in the cores of all the cheeses after 1, 10, 20, and 30 days of ripening, using a portable pH meter equipped with a Foodtrode electrode (Hamilton, Bonaduz, Switzerland) and a LabMaster-*a_w_* (Novasina AG, Switzerland), respectively. Concentrations of protein, fat, and salt; moisture contents; and pH and *a_w_* were average values from analyses performed on three samples coming from as many cheese-making trials. The total number of samples subjected to compositional analyses was 84, whereas 168 samples were subjected to determination of pH and *a_w_*.

Cell densities of presumptive mesophilic and thermophilic lactobacilli, mesophilic and thermophilic cocci, micrococci and staphylococci, enterococci, total coliforms, yeasts, and molds on the rinds and cores of all the cheeses after 1, 10, 20, and 30 days of ripening were determined as described previously (21). Twenty grams of cheese was homogenized with 180 ml sterile sodium citrate (2% [wt/vol]) solution, using a BagMixer 400 P (Interscience, St Nom, France) for 3 min of treatment. The enumeration of mesophilic and thermophilic lactobacilli and cocci was done at 30°C or 42°C, respectively, for 48 h on MRS and M17 agar (Oxoid) under anaerobiosis. Counting of micrococci and staphylococci and of enterococci was done at 37°C for 48 h on Baird Parker agar plus egg yolk tellurite and Slanetz-Bartley agar (Oxoid), respectively. The enumeration of total coliforms was done on violet red bile lactose agar (Oxoid) at 37°C for 24 h. Except for Slanetz-Bartley agar, the media for enumeration of bacteria were supplemented with cycloheximide at 0.1 g/liter. The number of yeast cells was estimated at 30°C for 48 h using Sabouraud dextrose agar (Oxoid) supplemented with chloramphenicol (0.1 g/liter). Mold enumeration was done on Wort agar (Oxoid) at 25°C for 5 days. Presumptive Le. lactis, V. casei, B. thermosphacta, and S. equorum cells were counted at 30°C for 24 h on MRS, Marine agar, *Corynebacterium* agar, and TSYE agar, respectively. Cell densities were calculated as average values of the results of analyses performed on three samples from as many cheese-making trials. Altogether, 168 samples were subjected to analyses of cultivable microbiota.

### Extraction of total bacterial DNA.

Surfaces and cores obtained from two samples of each cheese (CC and ATT, LL, VC, SE, BX, and MIX cheeses) after 1, 10, 20, and 30 days of ripening were subjected to extraction of bacterial DNA. Ninety milliliters of sterile saline solution was added to 10 g of cheese and homogenized for 5 min in a BagMixer 400 P. The homogenates were centrifuged (1,000 × *g*; 5 min; 4°C), and the supernatants were recovered and centrifuged (5,000 × *g*; 15 min; 4°C). The pellet was suspended in 0.5 ml of sterile saline solution, and the suspension was used for extraction of total DNA using a FastDNA spin kit for soil (MP Biomedicals, Solon, OH, USA) according to the manufacturer’s instructions. The concentration and purity of extracted DNA were assessed by spectrophotometric determination (Nanodrop ND-1000; Thermo Fisher Scientific Inc.).

### 16S rRNA gene amplicon library preparation, sequencing, and data analysis.

The DNA extracted from each part (surface and core) of each cheese after 1, 10, 20, and 30 days of ripening was used for PCR. The cheese microbiome was studied by sequencing of the V3-V4 region of the 16S rRNA gene, as previously described (49). Demultiplexed forward and reverse reads were joined using FLASH (50) and quality trimmed (Phred score, 20), and short reads (250 bp) were discarded using Prinseq (51). High-quality reads were then imported into QIIME 1 (52). OTUs were picked through a *de novo* approach using the clustering method UCLUST at a similarity threshold of 0.97 (12). Taxonomic assignment was obtained by using the RDP classifier and the Greengenes database (53), with a sequence similarity of 0.97. To avoid biases due to different sequencing depths, OTU tables were rarefied to the lowest number of sequences per sample. QIIME was used to calculate the alpha diversity (Chao1 richness and Shannon diversity indices) (54–56). PCoA, carried out in the R environment (https://www.r-project.org/), was based on the unweighted UniFrac distance matrix of all 16S rRNA gene sequences. The LEfSe algorithm (57) was used to identify the OTUs distinguishing cheeses with attenuated starter (alone or in combination with surface-inoculated bacteria) from the control cheese (58). The total number of samples subjected to 16S rRNA gene-sequencing analysis was 112.

### Assessment of proteolysis, enzyme activities, and concentrations of free amino acids.

Surfaces and cores obtained from three samples of each cheese (CC and ATT, LL, VC, SE, BX, and MIX cheeses) after 1, 10, 20, and 30 days of ripening were subjected to extraction of water-soluble fractions, as described by Kuchroo and Fox (59). The concentrations of peptides in the water-soluble extracts of the cheeses were determined by the *o*-phtaldialdehyde (OPA) method (60). A standard curve, prepared using tryptone (0.25 to 1.5 mg ml^−1^), was the reference. The use of peptone gave a similar standard curve. Enzyme activities (PepN, PepI, PepO, GDH, CGL, and esterase) from the water-soluble extracts of the cheeses were determined as described by Gobbetti et al. (61) and De Angelis et al. (62) (see the supplemental material for details). Total and individual FAAs from the water-soluble extracts were determined with a Biochrom series 30 amino acid analyzer (Biochrom Ltd., Cambridge Science Park, United Kingdom) as described by Siragusa et al. (63). The concentrations of peptides and FAAs and the enzyme activities were average values of the results of analyses performed on three samples from as many cheese-making trials. Altogether, 168 samples were subjected to analyses to determine the concentrations of peptides and FAAs and the enzyme activities. The concentrations of total and individual FAAs were elaborated through PCA, using the statistical software Statistica v. 7.0 for Windows.

### Sensory analysis.

Three panel sessions, one on each of three consecutive days, were held to evaluate the sensory characteristics of all the cheeses (CC and ATT, LL, VC, SE, BX, and MIX cheeses) after 30 days of ripening. The panel was compiled by 11 judges who had previously been trained in cheese profiling (64), with an equal distribution of males and females and ages ranging between 21 and 40 years. The definition of each sensory descriptor (see Table S3 in the supplemental material) was based on the studies by Niro et al. (65) and Albenzio et al. (66). For visual evaluation, the judge looked at the uniformity of crust and core colors (0, not uniform; 7, uniform) and the internal structure (0, uniform; 7, presence of holes). For evaluation by smelling, the judge smelled the wheel in different zones and evaluated the quality of odor sensation based on the intensity of the odor (0, none; 7, butter-like), rancid smell (0, none; 7, intense), and spicy smell (0, none; 7, intense). For evaluation by tasting, cheese samples, including rind and core, were coded with 3-digit randomized numbers and served at room temperature in aliquots (one per cheese variant) of 20 g, together with nonsalted table biscuits and still water, to panelists placed separately in rooms for impartial evaluation of sensory attributes. The judges evaluated the following characteristics: taste intensity (0, none; 5, aged), moisture (0, no; 5, too moist), acid (0, no acid; 5, too acid), bitter (0, not bitter; 5, too bitter), salty (0, not salty; 5, too salty), sweet (0, not sweet; 5, too sweet), and overall acceptability (0, dislike very much; 5, like very much). The total number of samples subjected to sensory analysis was 21.

### Statistical analyses.

A randomized complete-block split-plot design with three replicates for each cheese variant was used for the analyses. The only exception was for 16S rRNA metagenetic analysis, performed in duplicate. The data were subjected to one-way analysis of variance (ANOVA), and pair comparison of treatment means was achieved by Tukey’s procedure at a *P *value of <0.05, using Statistica v. 7.0. Multivariate differences among cheeses were estimated by PCA, using Statistica v. 7.0, and by the Permutational Multivariate Analysis of Variance Using Distance Matrices function of ADONIS (67). For ADONIS, distances among samples were first calculated using weighted UniFrac, and then an ANOVA-like simulation was conducted to test for group differences.

### Accession number(s).

The 16S rRNA gene sequences are available in the Sequence Read Archive of NCBI (BioProject no. PRJNA551421).

## Supplementary Material

Supplemental file 1

## References

[1] Fox PF, Uniacke-Lowe T, McSweeney PLH, O’Mahony JA (ed). 2015 Dairy chemistry and biochemistry, 2nd ed. Springer International Publishing, New York, NY.

[2] Khattab AR, Guirguis HA, Tawfik SM, Farag MA 2019 Cheese ripening: a review on modern technologies towards flavor enhancement, process acceleration and improved quality assessment. Trends Food Sci Technol 88:343–360. doi:10.1016/j.tifs.2019.03.009.

[3] Gobbetti M, De Angelis M, Di Cagno R, Mancini L, Fox PF 2015 Pros and cons for using non-starter lactic acid bacteria (NSLAB) as secondary/adjunct starters for cheese ripening. Trends Food Sci Technol 45:167–178. doi:10.1016/j.tifs.2015.07.016.

[4] Peralta GH, Bergamini CV, Hynes ER 2019 Disruption treatments on two strains of *Streptococcus thermophilus*: levels of lysis/permeabilisation of the cultures, and influence of treated cultures on the ripening profiles of Cremoso cheese. Int Dairy J 92:11–20. doi:10.1016/j.idairyj.2019.01.002.

[5] Doolan IA, Wilkinson MG 2009 Comparison of the effects of various attenuation methods on cell permeability and accessibility of intracellular enzymes in *Lactococcus lactis* strains. Int Dairy J 19:215–221. doi:10.1016/j.idairyj.2008.11.003.

[6] Di Cagno R, De Pasquale I, De Angelis M, Buchin S, Calasso M, Fox PF, Gobbetti M 2011 Manufacture of Italian caciotta-type cheeses with adjuncts and attenuated adjuncts of selected non-starter lactobacilli. Int Dairy J 21:254–260. doi:10.1016/j.idairyj.2010.12.007.

[7] Yarlagadda AB, Wilkinson MG, O'Sullivan MG, Kilcawley KN 2014 Utilisation of microfluidisation to enhance enzymatic and metabolic potential of lactococcal strains as adjuncts in Gouda type cheese. Int Dairy J 38:124–132. doi:10.1016/j.idairyj.2014.01.007.

[8] Montel MC, Buchin S, Mallet A, Delbes-Paus C, Vuitton DA, Desmasures N, Berthier F 2014 Traditional cheeses: rich and diverse microbiota with associated benefits. Int J Food Microbiol 177:136–154. doi:10.1016/j.ijfoodmicro.2014.02.019. 24642348

[9] Blaya J, Barzideh Z, LaPointe G 2018 Interaction of starter cultures and nonstarter lactic acid bacteria in the cheese environment. J Dairy Sci 101:3611–3629. doi:10.3168/jds.2017-13345. 29274982

[10] Gatti M, Bottari B, Lazzi C, Neviani E, Mucchetti G 2014 Microbial evolution in raw-milk, long-ripened cheeses produced using undefined natural whey starters. J Dairy Sci 97:573–591. doi:10.3168/jds.2013-7187. 24290824

[11] Stellato G, De Filippis F, La Storia A, Ercolini D 2015 Coexistence of lactic acid bacteria and potential spoilage microbiota in a dairy processing environment. Appl Environ Microbiol 81:7893–7904. doi:10.1128/AEM.02294-15. 26341209 PMC4616952

[12] De Filippis F, Genovese A, Ferranti P, Gilbert JA, Ercolini D, Gallo P 2016 Metatranscriptomics reveals temperature-driven functional changes in microbiome impacting cheese maturation rate. Sci Rep 6:21871. doi:10.1038/srep21871. 26911915 PMC4766472

[13] Gobbetti M, Di Cagno R, Calasso M, Neviani E, Fox PF, De Angelis M 2018 Drivers that establish and assemble the lactic acid bacteria biota in cheeses. Trends Food Sci Technol 78:244–254. doi:10.1016/j.tifs.2018.06.010.

[14] Kamimura BA, De Filippis F, Sant'Ana AS, Ercolini D 2019 Large-scale mapping of microbial diversity in artisanal Brazilian cheeses. Food Microbiol 80:40–49. doi:10.1016/j.fm.2018.12.014. 30704595

[15] Irlinger F, Mounier J 2009 Microbial interactions in cheese: implications for cheese quality and safety. Curr Opin Biotechnol 20:142–148. doi:10.1016/j.copbio.2009.02.016. 19342218

[16] Smid EJ, Kleerebezem M 2014 Production of aroma compounds in lactic fermentations. Annu Rev Food Sci Technol 5:313–326. doi:10.1146/annurev-food-030713-092339. 24580073

[17] Cocolin L, Mataragas M, Bourdichon F, Doulgeraki A, Pilet MF, Jagadeesan B, Rantsiou K, Phister T 2018 Next generation microbiological risk assessment meta-omics: the next need for integration. Int J Food Microbiol 287:10–17. doi:10.1016/j.ijfoodmicro.2017.11.008. 29157743

[18] Schmitz-Esser S, Dzieciol M, Nischler E, Schornsteiner E, Bereuter O, Mann E, Wagner M 2018 Abundance and potential contribution of Gram-negative cheese rind bacteria from Austrian artisanal hard cheeses. Int J Food Microbiol 266:95–103. doi:10.1016/j.ijfoodmicro.2017.11.013. 29190534

[19] Irlinger F, Layec S, Hélinck S, Dugat-Bony E 2015 Cheese rind microbial communities: diversity, composition and origin. FEMS Microbiol Lett 362:1–11. doi:10.1093/femsle/fnu015. 25670699

[20] Monnet C, Landaud S, Bonnarme P, Swennen D 2015 Growth and adaptation of microorganisms on the cheese surface. FEMS Microbiol Lett 362:1–9. doi:10.1093/femsle/fnu025. 25790503

[21] Calasso M, Ercolini D, Mancini L, Stellato G, Minervini F, Di Cagno R, De Angelis M, Gobbetti M 2016 Relationships among house, rind and core microbiotas during manufacture of traditional Italian cheeses at the same dairy plant. Food Microbiol 54:115–126. doi:10.1016/j.fm.2015.10.008.

[22] Salvadori del Prato O 2001 Caciotte, p 658–662. *In* Salvadori del Prato O (ed), Trattato di tecnologia casearia. Calderini Edagricole, Bologna, Italy.

[23] De Pasquale I, Di Cagno R, Buchin S, De Angelis M, Gobbetti M 2016 Spatial distribution of the metabolically active microbiota within Italian PDO ewes’ milk cheeses. PLoS One 11:e0153213. doi:10.1371/journal.pone.0153213. 27073835 PMC4830609

[24] Delgado S, Rachid C, Fernández E, Rychlik T, Alegría Á, Peixoto RS, Mayo B 2013 Diversity of thermophilic bacteria in raw, pasteurized and selectively-cultured milk, as assessed by culturing, PCR-DGGE and pyrosequencing. Food Microbiol 36:103–111. doi:10.1016/j.fm.2013.04.015. 23764225

[25] Aldrete-Tapia A, Escobar-Ramírez M, Tamplin M, Hernández-Iturriaga M 2014 High-throughput sequencing of microbial communities in Poro cheese, an artisanal Mexican cheese. Food Microbiol 44:136–141. doi:10.1016/j.fm.2014.05.022. 25084655

[26] De Pasquale I, Di Cagno R, Buchin S, De Angelis M, Gobbetti M 2019 Use of autochthonous mesophilic lactic acid bacteria as starter cultures for making Pecorino Crotonese cheese: effect on compositional, microbiological and biochemical attributes. Food Res Int 116:1344–1356. doi:10.1016/j.foodres.2018.10.024. 30716924

[27] Giannino ML, Marzotto M, Dellaglio F, Feligini M 2009 Study of microbial diversity in raw milk and fresh curd used for Fontina cheese production by culture independent methods. Int J Food Microbiol 130:188–195. doi:10.1016/j.ijfoodmicro.2009.01.022. 19232767

[28] Martins M, de Freitas MR, Deuvaux JC, Eller MR, Nero LA, de Carvalho AF 2018 Bacterial diversity of artisanal cheese from the Amazonian region of Brazil during the dry and rainy seasons. Food Res Int 108:295–300. doi:10.1016/j.foodres.2018.03.060. 29735061

[29] Fontana F, Cappa F, Rebecchi A, Cocconcelli PS 2010 Surface microbiota analysis of taleggio, gorgonzola, casera, scimudin and formaggio di fossa Italian cheeses. Int J Food Microbiol 138:205–211. doi:10.1016/j.ijfoodmicro.2010.01.017. 20167385

[30] Aldrete-Tapia A, Escobar-Ramírez CM, Tamplin ML, Hernández-Iturriaga M 2018 Characterization of bacterial communities in Mexican artisanal raw milk “bola de ocosingo” cheese by high-throughput sequencing. Front Microbiol 9:2598. doi:10.3389/fmicb.2018.02598. 30420851 PMC6217346

[31] Sánchez JI, Martínez B, Rodríguez A 2005 Rational selection of *Leuconostoc* strains for mixed starters based on the physiological biodiversity found in raw milk fermentations. Int J Food Microbiol 105:377–387. doi:10.1016/j.ijfoodmicro.2005.04.025. 16085331

[32] Bockelmann W 2007 Cheeses with secondary cultures: mould-ripened, smear-ripened, and farmhouse cheeses, p 494–519. In Weimer BC (ed), Improving the flavour of cheese, 1st ed Woodhead Publishing, Sawston, Cambridge, United Kingdom.

[33] Seixas FN, Rios EA, Martinez de Oliveira AL, Beloti V, Poveda JM 2018 Selection of *Leuconostoc* strains isolated from artisanal Serrano Catarinense cheese for use as adjuncts in cheese manufacture. J Sci Food Agric 98:3899–3906. doi:10.1002/jsfa.8907. 29364508

[34] Ramezani M, Hosseini SM, Ferrocino I, Amoozegar MA, Cocolin L 2017 Molecular investigation of bacterial communities during the manufacturing and ripening of semi-hard Iranian Liqvan cheese. Food Microbiol 66:64–71. doi:10.1016/j.fm.2017.03.019. 28576374

[35] Kastman EK, Kamelamela N, Norville JW, Cosetta CM, Dutton RJ, Wolfe BE 2016 Biotic interactions shape the ecological distributions of *Staphylococcus* species. mBio 7:e01157-16. doi:10.1128/mBio.01157-16. 27795388 PMC5082897

[36] Coton E, Desmonts MH, Leroy S, Coton M, Jamet E, Christieans S, Donnio PY, Lebert I, Talon R 2010 Biodiversity of coagulase-negative staphylococci in French cheeses, dry fermented sausages, processing environments and clinical samples. Int J Food Microbiol 137:221–229. doi:10.1016/j.ijfoodmicro.2009.11.023. 20061042

[37] Yeluri Jonnala BR, McSweeney PLH, Sheehan JJ, Cotter PD 2018 Sequencing of the cheese microbiome and its relevance to industry. Front Microbiol 9:1020. doi:10.3389/fmicb.2018.01020. 29875744 PMC5974213

[38] Gaglio R, Scatassa ML, Cruciata M, Miraglia V, Corona O, Di Gerlando R, Portolano B, Moschetti G, Settanni L 2014 In vivo application and dynamics of lactic acid bacteria for the four-season production of Vastedda-like cheese. Int J Food Microbiol 177:37–48. doi:10.1016/j.ijfoodmicro.2014.02.007. 24598514

[39] Silvetti T, Capra E, Morandi E, Cremonesi P, Decimo M, Gavazzi F, Giannico R, De Noni I, Brasca M 2017 Microbial population profile during ripening of protected designation of origin (PDO) Silter cheese produced with and without autochthonous starter culture. Lebenson Wiss Technol 84:821–831. doi:10.1016/j.lwt.2017.06.022.

[40] Dugat-Bony E, Straub C, Teissandier A, Onésime D, Loux V, Monnet C, Irlinger F, Landaud S, Leclercq-Perlat M-N, Bento P, Fraud S, Gibrat J-F, Aubert J, Fer F, Guédon E, Pons N, Kennedy S, Beckerich J-M, Swennen D, Bonnarme P 2015 Overview of a surface-ripened cheese community functioning by meta-omics analyses. PLoS One 10:e0124360. doi:10.1371/journal.pone.0124360. 25867897 PMC4395090

[41] Grant IR, Hitchings EI, McCartney A, Ferguson F, Rowe MT 2002 Effect of commercial-scale high-temperature, short-time pasteurization on the viability of *Mycobacterium paratuberculosis* in naturally infected cows’ milk. Appl Environ Microbiol 68:602–607. doi:10.1128/aem.68.2.602-607.2002. 11823197 PMC126679

[42] Keklik NM, Elik A, Salgin U, Demirci A, Koçer G 2019 Inactivation of *Staphylococcus aureus* and *Escherichia coli* O157:H7 on fresh kashar cheese with pulsed ultraviolet light. Food Sci Technol Int 25:680–691. doi:10.1177/1082013219860925. 31272222

[43] Carminati D, Perrone A, Neviani E, Mucchetti G 2000 Influence of traditional brine washing of smear Taleggio cheese on the surface spreading of *Listeria innocua*. J Food Prot 63:1353–1358. doi:10.4315/0362-028x-63.10.1353. 11041134

[44] Goerges S, Mounier J, Rea MC, Gelsomino R, Heise V, Beduhn R, Cogan TM, Vancanneyt M, Scherer S 2008 Commercial ripening starter microorganisms inoculated into cheese milk do not successfully establish themselves in the resident microbial ripening consortia of a South German red smear cheese. Appl Environ Microbiol 74:2210–2217. doi:10.1128/AEM.01663-07. 18281427 PMC2292584

[45] International Dairy Federation. 1964 Determination of the protein content of processed cheeses products. Standard 25. International Dairy Federation, Brussels, Belgium.

[46] Institute for Industrial Research and Standards. 1955 Determination of the percentage of fat in cheese. Irish standard 69. Institute for Industrial Research and Standards, Dublin, Ireland.

[47] International Dairy Federation. 1982 Cheese and processed cheese. Determination of the total solid content. Standard 4A. International Dairy Federation, Brussels, Belgium.

[48] Fox PF 1963 Potentiometric determination of salt in cheese. J Dairy Sci 46:744–745. doi:10.3168/jds.S0022-0302(63)89134-1.

[49] Berni Canani R, De Filippis F, Nocerino R, Laiola M, Paparo L, Calignano A, De Caro C, Coretti L, Chiariotti L, Gilbert JA, Ercolini D 2017 Specific signatures of the gut microbiota and increased levels of butyrate in children treated with fermented cow’s milk containing heat-killed *Lactobacillus paracasei* CBA L74. Appl Environ Microbiol 83:e01206-17. doi:10.1128/AEM.01206-17. 28733284 PMC5601345

[50] Magoc T, Salzberg SL 2011 FLASH: fast length adjustment of short reads to improve genome assemblies. Bioinformatics 27:2957–2963. doi:10.1093/bioinformatics/btr507. 21903629 PMC3198573

[51] Schmieder R, Edwards R 2011 Quality control and preprocessing of metagenomic datasets. Bioinformatics 27:863–864. doi:10.1093/bioinformatics/btr026. 21278185 PMC3051327

[52] Caporaso JG, Kuczynski J, Stombaugh J, Bittinger K, Bushman FD, Costello EK, Fierer N, Peña AG, Goodrich JK, Gordon JI, Huttley GA, Kelley ST, Knights D, Koenig JE, Ley RE, Lozupone CA, McDonald D, Muegge BD, Pirrung M, Reeder J, Sevinsky JR, Turnbaugh PJ, Walters WA, Widmann J, Yatsunenko T, Zaneveld J, Knight R 2010 QIIME allows analysis of high-throughput community sequencing data. Nat Methods 7:335–336. doi:10.1038/nmeth.f.303. 20383131 PMC3156573

[53] McDonald D, Price MN, Goodrich J, Nawrocki EP, DeSantis TZ, Probst A, Andersen GL, Knight R, Hugenholtz P 2012 An improved Greengenes taxonomy with explicit ranks for ecological and evolutionary analyses of bacteria and archea. ISME J 6:610–618. doi:10.1038/ismej.2011.139. 22134646 PMC3280142

[54] Chao A, Bunge J 2002 Estimating the number of species in a stochastic abundance model. Biometrics 58:531–539. doi:10.1111/j.0006-341x.2002.00531.x. 12229987

[55] Shannon CE, Weaver W 1949 The mathematical theory of communication. University of Illinois Press, Urbana, IL.

[56] Suchodolski JS, Dowd SE, Wilke V, Steiner JM, Jergens AE 2012 16S rRNA gene pyrosequencing reveals bacterial dysbiosis in the duodenum of dogs with idiopathic inflammatory bowel disease. PLoS One 7:e39333. doi:10.1371/journal.pone.0039333. 22720094 PMC3376104

[57] Afgan E, Baker D, Batut B, van den Beek M, Bouvier D, Čech M, Chilton J, Clements D, Coraor N, Grüning BA, Guerler A, Hillman-Jackson J, Hiltemann S, Jalili V, Rasche H, Soranzo N, Goecks J, Taylor J, Nekrutenko A, Blankenberg D 2018 The Galaxy platform for accessible, reproducible and collaborative biomedical analyses: 2018 update. Nucleic Acids Res 46:W537–W544. doi:10.1093/nar/gky379. 29790989 PMC6030816

[58] Segata N, Izard J, Waldron L, Gevers D, Miropolsky L, Garrett WS, Huttenhower C 2011 Metagenomic biomarker discovery and explanation. Genome Biol 12:R60. doi:10.1186/gb-2011-12-6-r60. 21702898 PMC3218848

[59] Kuchroo CN, Fox PF 1982 Soluble nitrogen in Cheddar cheese: comparison of extraction procedures. Milchwissenschaft 37:331–335.

[60] Church FC, Swaisgood HE, Porter DH, Catignani GL 1983 Spectrophotometric assay using o-phthaldialdehyde for determination of proteolysis in milk and isolated milk proteins. J Dairy Sci 66:1219–1227. doi:10.3168/jds.S0022-0302(83)81926-2.

[61] Gobbetti M, Folkertsma B, Fox PF, Corsetti A, Smacchi E, De Angelis M, Rossi J, Kilcawley K, Cortini M 1999 Microbiology and biochemistry of Fossa (pit) cheese. Int Dairy J 9:763–773. doi:10.1016/S0958-6946(99)00147-8.

[62] De Angelis M, Calasso M, Di Cagno R, Minervini F, Gobbetti M 2010 NADP-glutamate dehydrogenase activity in non-starter lactic acid bacteria: effects of temperature, pH and NaCl on enzyme activity and expression. J Appl Microbiol 109:1763–1774. doi:10.1111/j.1365-2672.2010.04804.x. 20662973

[63] Siragusa S, De Angelis M, Di Cagno R, Rizzello CG, Coda R, Gobbetti M 2007 Synthesis of γ-aminobutyric acid by lactic acid bacteria isolated from a variety of Italian cheeses. Appl Environ Microbiol 73:7283–7290. doi:10.1128/AEM.01064-07. 17890341 PMC2168214

[64] Faccia M, Mastromatteo M, Conte A, Del Nobile MA 2013 Influence of the milk bactofugation and natural whey culture on the microbiological and physicochemical characteristics of mozzarella. J Food Process Technol 4:4. doi:10.4172/2157-7110.1000218.

[65] Niro S, Fratianni A, Tremonte P, Sorrentino E, Tipaldi L, Panfili G, Coppola R 2014 Innovative Caciocavallo cheeses made from a mixture of cow milk with ewe or goat milk. J Dairy Sci 97:1296–1304. doi:10.3168/jds.2013-7550. 24440270

[66] Albenzio M, Santillo A, Caroprese M, Braghieri A, Sevi A, Napolitano F 2013 Composition and sensory profiling of probiotic Scamorza ewe milk cheese. J Dairy Sci 96:2792–2800. doi:10.3168/jds.2012-6273. 23522674

[67] Oksanen J, Blanchet FG, Friendly M, Kindt R, Legendre P, McGlinn D, Minchin PR, O’Hara RB, Simpson GL, Solymos P, Stevens MHH, Szoecs E, Wagner H 2016 Vegan: community ecology package. R package version 2-4-0. https://cran.r-project.org/web/packages/vegan/index.html.

