# Supplementary material for: Attenuated Lactococcus lactis and Surface Bacteria as Tools for Conditioning the Microbiota and Driving the Ripening of Semisoft Caciotta Cheese
Source: Appl Environ Microbiol. 2020 Feb 18;86(5):e02165-19. doi: 10.1128/AEM.02165-19 (PMC7028956; doi:10.1128/AEM.02165-19)
Supplement: Supplemental file 1 [file AEM.02165-19-s0001.pdf]

RESULTS

Kinetics of growth and enzymatic activities. *Leuc. lactis* MC11, *V. casei* DSM22364, *St. equorum* DSM15097 and *B. thermosphacta* MC25 were representatives of the surface microbiota of Caciotta cheese (21, 69, 70). Preliminarily, the capability of each strain to grow in cheese matrix was assessed using cheese broth (CB) as model system (71). To simulate the cheese surface contamination, the initial inoculum was 2.0 log CFU/ml. Inoculated CB was held at room temperature (25 °C) for 12 h, with a further stay at 9 °C for 10 days. All strains reached the stationary phase after 7 days. The final cell density varied from 8.1 ± 0.12 (*B. thermosphacta* MC25) to 8.6 ± 0.07 (*Leuc. lactis* MC11) log CFU/ml (data not shown).

Preliminarily, aminopeptidase type N (pepN), proline iminopeptidase (pepI), endopeptidase type O (pepO), glutamate dehydrogenase (GDH), cystathionine gamma lyase (CGL) and esterase activities of *Lc. lactis* subsp. *lactis* CC01, *Leuc. lactis* MC11, *V. casei* DSM22364, *St. equorum* DSM15097 and *B. thermosphacta* MC25 were assessed on synthetic substrates (Table S1). Except for pepI and pepO, which were the highest for attenuated *Lc. lactis* CC01, all the other enzyme activities were at the highest levels in *Leuc. lactis* and *B. thermosphacta*. Nevertheless, all the strains assessed showed remarkable enzyme activities.

Table S1. Enzymatic activities[1] of *Lactococcus lactis* subsp. *lactis* CC1 (LCL), *Leuconostoc lactis* MC11 (LL), *Vibrio casei* DSM22364 (VC), *Staphylococcus equorum* DSM15097 (SE), *Brochothrix thermosphacta* MC25 (BX).

**Enzymes**	**LCL**	**LL**	**VC**	**SE**	**BX**
Aminopeptidase type N	15.8±0.45B	26±0.94A	3.4±0.13C	2.7±0.08C	13±0.52B
Proline iminopeptidase	16.6±0.68A	5.9±0.22AB	2.16±0.09B	6.3±0.24AB	7.9±0.31A
Endopeptidase type O	8.3±0.26A	1.8±0.06B	1.9±0.04B	0	2.2±0.09B
Glutamate dehydrogenase	0.4±0.01B	1.8±0.05A	0.5±0.02B	0	0.5±0.01B
Cystathionine gamma-lyase	0	16.5±0.31A	0	0	11.2±0.17B
Esterase	0	3.88±0.15A	1.5±0.06B	0	4.21±0.12A

Data in the same row with different letters (A-C) are significantly different ($P < 0.05$).

[1]Arbitrary units (see Materials and Methods). Values represent the average (± SD) of 3 triplicates.

Table S2. Concentrations (mg/kg) of total and individual free amino acids and of amino acid derivatives at surface (s) and core (c) level of control cheese (CC), cheese added of attenuated culture of *Lactococcus lactis* subsp. *lactis* CC1 (ATT) and ATT cheeses with the additions, as surface adjunct cultures (2 log CFU/cm^2), of *Leuconostoc lactis* MC11 (LL), *Vibrio casei* DSM22364 (VC), *Staphylococcus equorum* DSM15097 (SE), *Brochothrix thermosphacta* MC25 (BX) individually or in mix (MIX) after 1, 10, 20 or 30 days of ripening.

1 day of ripening

FAA	CCs	ATTs	LLs	VCs	SEs	BXs	MIXs	CCc	ATTc	LLc	VCc	SEc	BXc	MIXc
**Asp**	24.5C	53.4B	47.2BC	88.8A	57.0B	82.4A	77.1AB	6.3D	9.6D	41.2BC	43.3BC	39.8BC	34.3BC	34.9BC
**Thr**	15.4B	41.1AB	38.9AB	57.7A	13.6B	56.6A	46.0AB	0.0	17.4B	47.4AB	48.0AB	47.1AB	34.4AB	42.6ABA
**Ser**	37.1C	95.5BC	144.4B	209.6A	49.0C	207.2A	147.2B	47.8C	52.4C	175.5AB	180.7AB	179.0AB	127.8B	168.2AB
**Glu**	108.3C	284.0B	396.4A	409.8A	291.5B	438.8A	212.9B	33.5D	98.9D	266.0B	262.9B	258.1B	211.7B	242.1B
**Gly**	3.8B	16.9AB	23.1A	31.2A	15.4AB	25.3A	19.6AB	4.9B	6.7B	19.3AB	19.3AB	19.2AB	13.6AB	19.1AB
**Ala**	54.1BC	87.1B	133.8AB	173.6A	93.1B	149.7A	162.7A	23.1C	28.2C	74.6B	73.2B	72.4B	52.9BC	67.4B
**Cys**	20.3B	25.9B	36.5AB	52.4A	29.7B	58.5A	40.6AB	6.8C	7.5C	12.6C	0.0	13.6C	0.0	12.9C
**Val**	107.5CD	240.7B	302.3AB	337.8A	254.8B	349.5A	284.2B	27.9E	66.2D	170.3C	176.4C	168.2C	124.4CD	155.6C
**Met**	73.1C	104.5B	141.1AB	174.1A	107.3B	179.2A	69.3C	18.0D	34.2CD	87.6C	88.4C	87.7C	46.4CD	82.8C
**Ile**	49.0CD	130.3B	196.3AB	249.6A	143.9B	240.8A	191.6AB	11.2D	82.4C	88.3C	89.0C	87.4C	55.5CD	83.0C
**Leu**	178.8E	359.7C	468.4B	505.4AB	384.6C	517.8AB	581.1A	283.7D	237.5D	332.9C	335.6C	334.3C	259.0D	317.4C
**Tyr**	80.5C	91.6C	138.8B	190.4A	117.1B	172.1A	169.3A	39.3D	25.9D	66.0CD	70.6CD	68.8CD	41.7D	69.3CD
**Phe**	168.6C	259.1B	378.7AB	428.8A	281.1B	450.7A	385.8AB	65.2D	77.5D	199.3BC	195.6BC	211.0BC	149.0C	199.3BC
**His**	60.5C	56.9C	105.3A	113.3A	59.4C	95.2AB	84.4B	24.0D	19.8D	48.9C	50.3C	51.6C	39.0CD	46.8C
**Trp**	17.7C	60.3B	84.1AB	101.9A	60.2B	106.7A	80.6AB	11.8C	13.5C	45.1BC	51.4BC	51.3BC	30.5BC	48.8BC
**Lys**	101.6BC	146.5B	216.6A	280.7A	180.9AB	248.0A	227.6A	35.9C	51.0C	99.6BC	105.9BC	117.1BC	75.0C	104.5BC
**Arg**	126.6A	37.4C	34.4C	54.3BC	27.1C	37.0C	36.6C	71.1B	3.8D	7.9D	8.6D	8.9D	6.8D	9.4D
**Pro**	88.8D	186.1C	298.2B	345.7A	221.6B	354.9A	263.8B	120.9CD	182.4C	164.9C	174.5C	176.3C	137.7C	163.8C
**Total**	1316.1C	2277.0B	3184.5AB	3805.1A	2387.2B	3770.4A	3080.4AB	831.5D	1014.9	1947.4BC	1973.9BC	1991.5BC	1439.6C	1867.9BC
**GABA**	138.1B	72.0BC	102.2B	98.2B	106.0B	242.6A	27.1CD	44.5C	17.1D	45.9C	50.2C	49.7C	30.7CD	44.4C
**Orn**	15.6C	34.0AB	58.0A	62.0A	36.7AB	68.7A	25.3B	6.5C	10.7C	41.1AB	47.4AB	46.0AB	32.4B	32.3B
**Amm**	154.3D	294.8B	409.1A	416.4A	260.3C	395.8A	234.5C	101.3D	133.0D	276.4BC	308.7B	273.4BC	239.1C	239.9C

28

10 days of ripening														
**FAA**	**CCs**	**ATTs**	**LLs**	**VCs**	**SEs**	**BXs**	**MIXs**	**CCc**	**ATTc**	**LLc**	**VCc**	**SEc**	**BXc**	**MIXc**
**Asp**	59.4B	65.5AB	80.5A	64.6AB	56.2AB	65.1AB	65.7AB	20.7C	84.7A	90.8A	96.7A	85.5A	78.4A	95.9A
**Thr**	26.1C	66.6BC	86.8B	75.7BC	68.6BC	63.7BC	56.3BC	18.9C	92.5B	104.2AB	112.1AB	100.2AB	89.2B	120.1A
**Ser**	185.7D	253.0C	319.9B	286.5C	254.6C	252.1C	258.6C	167.5D	388.4AB	433.5A	458.9A	408.4AB	385.1AB	451.1A
**Glu**	230.0D	345.4C	403.5B	363.4C	332.2C	508.9A	462.2B	83.7E	381.3C	402.3B	417.5B	395.2C	378.3C	438.1B
**Gly**	15.9C	35.9BC	44.3B	40.5B	40.0B	37.2B	48.9B	33.0BC	73.3AB	71.9AB	77.9AB	75.4AB	70.2AB	85.5A
**Ala**	57.6C	91.1B	115.6AB	94.8B	83.6B	89.6B	82.9B	62.7BC	115.5AB	123.0AB	127.6AB	119.8AB	116.0AB	141.6A
**Cys**	29.8B	40.9AB	51.0A	40.2AB	30.2B	43.5AB	29.6B	13.5C	22.9B	22.1B	23.7B	24.7B	22.2B	24.2B
**Val**	185.8BC	274.6A	314.2A	286.9A	221.1B	290.5A	271.1AB	108.9C	238.0AB	250.9AB	257.8AB	243.8AB	239.6AB	269.1AB
**Met**	75.3B	121.5AB	153.3AB	129.1AB	102.1B	124.4AB	154.4AB	95.6B	168.3A	175.9A	189.2A	181.7A	164.8A	190.3A
**Ile**	67.9C	178.1AB	219.8A	174.9AB	124.9B	168.4AB	132.7B	40.3BC	127.8B	142.8AB	159.7AB	141.2AB	128.4B	159.4AB
**Leu**	221.5C	441.7AB	487.5AB	458.8AB	392.8B	460.9AB	447.1AB	289.9BC	480.4A	489.2A	510.6A	498.4A	473.2A	512.4A
**Tyr**	87.3AB	94.6A	103.2A	74.4C	41.0D	95.1A	73.0C	94.5A	64.3C	75.2C	88.3AB	74.1C	63.9C	69.7C
**Phe**	205.3C	351.4B	413.9AB	376.3B	289.0BC	389.7AB	359.2B	243.9C	417.9AB	433.4A	448.6A	431.3AAB	407.9AB	450.9A
**His**	90.1B	100.6AB	130.8A	89.1B	73.1BC	100.6AB	93.5B	60.7C	76.3BC	77.5BC	81.5B	75.1BC	77.4BC	84.5B
**Trp**	55.2B	75.0AB	94.1A	85.5AB	54.2B	91.2A	85.6AB	52.9B	78.5AB	67.0AB	87.2AB	85.1AB	76.1AB	83.4A
**Lys**	156.4B	219.0AB	260.1A	211.1AB	168.8B	196.3AB	171.9B	89.8C	155.8B	179.9AB	184.4AB	168.6AB	157.5B	203.9AB
**Arg**	142.3B	49.4C	67.1BC	36.8C	29.9C	57.1BC	60.4BC	201.8A	39.1C	38.6C	39.5C	35.0C	40.4C	40.4C
**Pro**	57.4D	238.6AB	273.0AB	243.5AB	211.4B	222.4B	148.1C	70.4D	273.8AB	297.2AB	304.1A	285.4AB	262.2AB	328.5A
**Total**	1949.0D	3043.0B	3618.5AB	3132.2B	2573.7C	3256.6B	3051.4B	1748.5D	3278.9B	3475.4AB	3665.3A	3428.9AB	3230.9B	3748.9A
**GABA**	164.0AB	178.7AB	274.1A	183.6AB	100.0B	205.7A	230.0A	178.1AB	143.7AB	124.5B	136.2AB	145.8AB	122.7AB	132.8AB
**Orn**	18.8C	39.4B	49.7B	58.1B	44.5B	57.5B	42.8B	16.2C	76.2AB	64.2AB	80.8AB	81.5AB	68.6AB	92.5A
**Amm**	94.2C	185.5B	224.3B	194.2B	206.0B	174.9B	169.8B	155.4B	317.7AB	329.8AB	328.4AB	339.9AB	311.1AB	385.4A

20 days of ripening														
**FAA**	**CCs**	**ATTs**	**LLs**	**VCs**	**SEs**	**BXs**	**MIXs**	**CCc**	**ATTc**	**LLc**	**VCc**	**SEc**	**BXc**	**MIXc**
**Asp**	44.6BC	37.1BC	74.2AB	50.6B	38.8BC	13.5C	53.8B	26.4BC	87.7AB	102.1A	71.0AB	103.9A	37.5BC	85.4AB
**Thr**	68.5A	35.6B	37.6B	48.8AB	71.9A	12.6C	74.9A	34.4B	69.6A	83.8A	74.6A	73.5A	36.4B	86.2A
**Ser**	696.4CD	677.3CD	1052.0C	1049.4C	1221.4BC	402.2D	1471.9BC	865.7CD	1831.1B	2369.5A	2110.8AB	2009.8AB	1025.2C	2421.3A
**Glu**	518.2A	473.0AB	577.0A	514.1A	512.0A	125.9C	550.9A	231.0B	522.2A	612.2A	514.1A	572.6A	285.8B	554.0A
**Gly**	4.5B	5.0B	16.8AB	7.2B	6.5B	4.3B	14.1AB	5.2B	19.9AB	34.9A	23.9AB	19.7AB	10.6B	32.5A
**Ala**	52.2B	49.2B	89.3AB	65.3B	72.1AB	48.7B	87.2AB	48.0B	103.0AB	128.2A	114.8AB	103.8AB	60.8B	128.1A
**Cys**	19.3	20.5	25.7	21.7	20.9	15.4	23.5	13.6	24.4	25.0	19.7	21.3	15.2	21.8
**Val**	216.8A	226.9A	278.5A	253.2A	267.4A	263.7A	287.3A	160.5B	241.1A	298.2A	238.2A	253.7A	131.1B	274.7A
**Met**	101.4AB	110.3A	128.1A	127.4A	137.6A	37.6B	145.1A	61.9B	129.6A	155.0A	125.4A	132.7A	60.2B	144.3A
**Ile**	91.2AB	115.4AB	139.2A	130.3A	148.9A	25.8B	166.3A	37.6B	136.2A	174.7A	146.0A	147.5A	71.3AB	175.0A
**Leu**	364.4C	422.8B	488.6AB	470.3B	488.5AB	458.0B	518.2AB	199.8D	527.9AB	618.7A	507.3AB	559.6AB	284.8CD	582.9A
**Tyr**	100.6AB	92.0BC	102.0AB	111.7AB	126.4A	75.4BC	119.8A	82.2B	107.9AB	103.7AB	112.2AB	113.1AB	60.4C	124.7A
**Phe**	161.7B	236.9AB	289.4A	298.0A	291.2A	124.9B	307.3A	123.2B	283.8AB	331.8A	238.3AB	275.2AB	141.1B	297.8A
**His**	13.7C	20.5BC	48.8AB	30.9B	21.7B	34.0B	50.3AB	29.9B	73.0A	79.6A	66.6A	79.5A	47.9AB	56.1AB
**Trp**	27.4B	63.5AB	68.6AB	80.1A	75.7A	30.7B	76.7A	27.0C	45.7AB	80.8A	54.9AB	65.3AB	32.7AB	75.6A
**Lys**	29.0C	29.3C	96.1B	43.1BC	50.9BC	90.7B	117.5AB	82.2BC	60.6BC	131.3AB	113.8AB	78.7BC	66.4BC	161.3A
**Arg**	4.7	1.9	5.1	5.8	9.7	106.0	7.7	1.1	10.3	8.8	16.0	12.4	6.9	14.6
**Pro**	121.2B	284.1AB	279.1AB	288.0AB	319.7A	383.0A	339.3A	74.4C	245.0AB	302.5AB	228.7AB	233.3AB	125.4B	276.8AB
**Total**	2636.0D	2901.2D	3796.2C	3596.0C	3881.1	3442.3C	4412.0B	2104.2D	4497.9B	5640.8A	4776.1B	4855.5B	2499.7D	5513.2A
**GABA**	147.7B	225.0A	145.1B	136.4B	136.8B	132.9B	141.0B	197.6AB	166.6AB	131.6B	158.0AB	151.8AB	139.3B	134.2B
**Orn**	24.4B	29.0B	53.2AB	35.8B	41.8AB	9.0C	59.0AB	4.4C	65.1AB	91.4A	55.2AB	66.5AB	34.9B	74.9AB
**Amm**	156.6A	142.6AB	170.1A	162.2A	159.1A	66.1C	164.8A	61.3C	162.8A	180.3A	153.4A	169.6A	105.1B	166.8A

30 days of ripening														
**FAA**	**CCs**	**ATTs**	**LLs**	**VCs**	**SEs**	**BXs**	**MIXs**	**CCc**	**ATTc**	**LLc**	**VCc**	**SEc**	**BXc**	**MIXc**
**Asp**	89.6BC	166.6A	126.9AB	134.1AB	152.9A	133.9AB	112.0AB	46.2C	90.3BC	80.0BC	112.2AB	86.9BC	105.6B	105.5B
**Thr**	98.2B	148.4A	162.2A	138.4AB	126.1AB	107.9AB	172.2A	88.1B	106.5AB	100.5AB	120.8AB	99.8AB	115.1AB	99.5AB
**Ser**	170.3C	306.4B	405.6A	348.5AB	311.0B	271.7BC	390.3AB	165.6C	323.0AB	289.5AB	361.7AB	296.8AB	339.9AB	389.4AB
**Glu**	141.5C	112.3C	450.8A	377.5A	346.1AB	318.0AB	396.5A	134.0C	299.4AB	261.0B	330.2AB	265.7B	311.2AB	251.7B
**Gly**	46.4AB	103.9A	63.8AB	50.7AB	51.1AB	51.0AB	62.3AB	33.5B	58.6AB	55.9AB	64.0AB	55.5AB	65.1AB	54.1AB
**Ala**	151.3B	317.1A	219.0AB	184.6AB	167.8AB	164.9AB	201.3AB	85.8C	139.2B	122.7B	147.4AB	122.2B	146.3AB	115.5B
**Cys**	46.3BC	178.0A	92.3B	70.2AB	84.4AB	75.4AB	58.7AB	23.5C	30.1C	25.7C	31.2C	27.9C	36.1BC	25.9C
**Val**	202.0B	377.3AB	487.2A	482.6A	448.1A	454.2A	457.7A	198.8B	303.3AB	256.5B	323.8AB	262.0B	313.9AB	244.7B
**Met**	174.7BC	426.8A	302.7AB	269.6B	250.1B	234.2B	252.6B	152.7C	223.4B	174.2BC	236.3B	187.7BC	235.5B	271.7B
**Ile**	191.0C	430.9A	431.8A	390.3AB	351.6B	336.5B	370.0B	126.5D	185.4C	151.4BC	201.8C	158.4BC	185.6C	147.8BC
**Leu**	525.8CD	637.5C	1000.1A	926.1AB	887.2B	859.6B	870.9B	327.5D	769.2BC	640.3C	818.5B	681.6BC	758.6BC	631.3C
**Tyr**	90.0B	99.4B	131.6AB	177.9A	195.0A	194.8A	158.3AB	179.9	103.4B	64.8C	109.5B	73.9C	80.3BC	70.2C
**Phe**	344.0D	364.3D	846.3A	709.4AB	666.4B	650.0B	644.0B	272.6E	510.5C	397.3D	560.4C	432.6CD	514.3C	594.2C
**His**	91.8B	85.4BC	160.9A	179.6A	64.9C	143.9AB	120.1AB	109.9B	102.7B	82.0BC	109.0B	91.2B	106.2B	81.9BC
**Trp**	65.5C	126.4AB	191.6A	128.3AB	158.5AB	152.4AB	133.9AB	85.8BC	106.1B	66.6C	111.5B	87.9BC	107.8B	72.7C
**Lys**	240.6B	444.3A	273.3B	431.5A	411.2AB	394.2AB	459.6A	134.8C	213.1B	188.6BC	229.9B	192.3BC	221.2B	187.6BC
**Arg**	77.9AB	57.3B	19.4C	66.7AB	97.6A	89.2A	88.7A	39.6BC	43.0BC	36.0BC	38.9BC	40.4BC	47.0BC	57.3AB
**Pro**	170.6CD	248.7C	506.2A	427.8B	378.7BC	353.9BC	430.7B	54.7D	352.3BC	294.2C	367.8BC	302.7C	351.2BC	294.3C
**Total**	2917.4D	4100.9C	5871.5A	5493.8AB	5148.7AB	4985.5B	5379.9AB	2259.5E	3959.5C	3287.2D	4274.7BC	3465.6BC	4040.9C	3695.1BC
**GABA**	97.8C	473.8A	155.1BC	127.3C	149.1BC	127.0C	121.9C	271.3B	158.6BC	125.5C	182.9BC	147.0BC	170.2BC	133.1C
**Orn**	15.7C	191.2A	158.7AB	91.7B	86.7B	97.5B	82.5B	29.5C	7.1C	57.4BC	103.7B	71.9BC	92.3B	1.9C
**Amm**	55.5B	233.7A	229.9A	185.0AB	160.0AB	161.7AB	174.2AB	78.3B	166.2AB	158.8AB	190.0AB	151.0AB	174.7AB	153.9AB

Values represent the average of analysis performed on three samples coming from as many cheese-making experiments. Data in the same row with different letters (A-E) are significantly different (*P* <0.05).

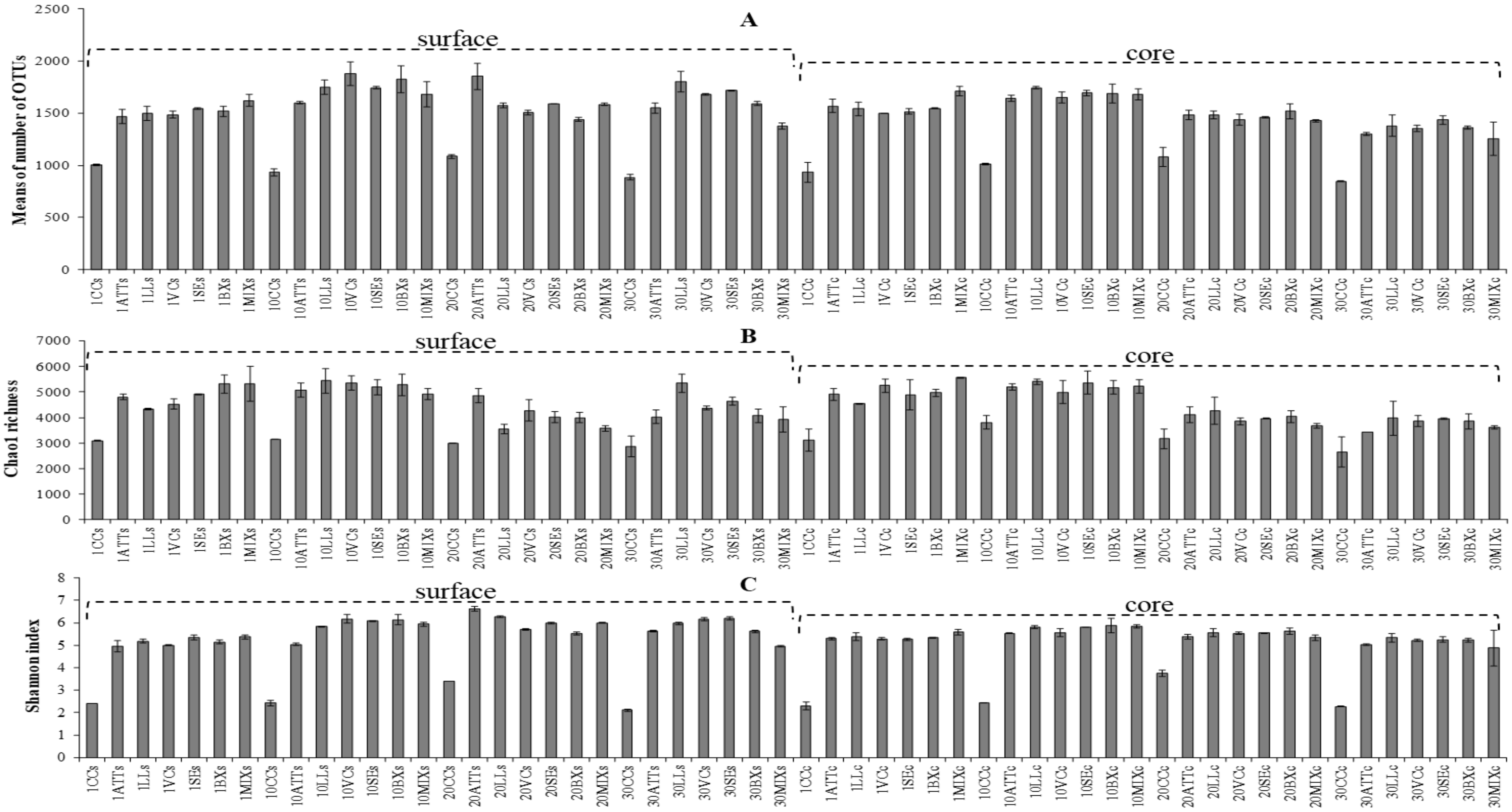

Figure S1. Number of bacterial Operational Taxonomic Units (OTUs) (A), Chao1 richness (B); and Shannon index (C) found upon 16S metagenetic analysis performed using the DNA extracted from surface (s) and core (c) level of control cheese (CC), cheese added of attenuated culture of *Lactococcus lactis* subsp. *lactis* CC01 (ATT) and ATT cheeses with the additions, as surface adjunct cultures (2 log CFU/cm^2), of *Leuconostoc lactis*

MC11 (LL), *Vibrio casei* DSM22364 (VC), *Staphylococcus equorum* DSM15097 (SE), *Brochothrix thermosphacta* MC25 (BX) individually or in mix (MIX) after 1, 10, 20 or 30 days of ripening.

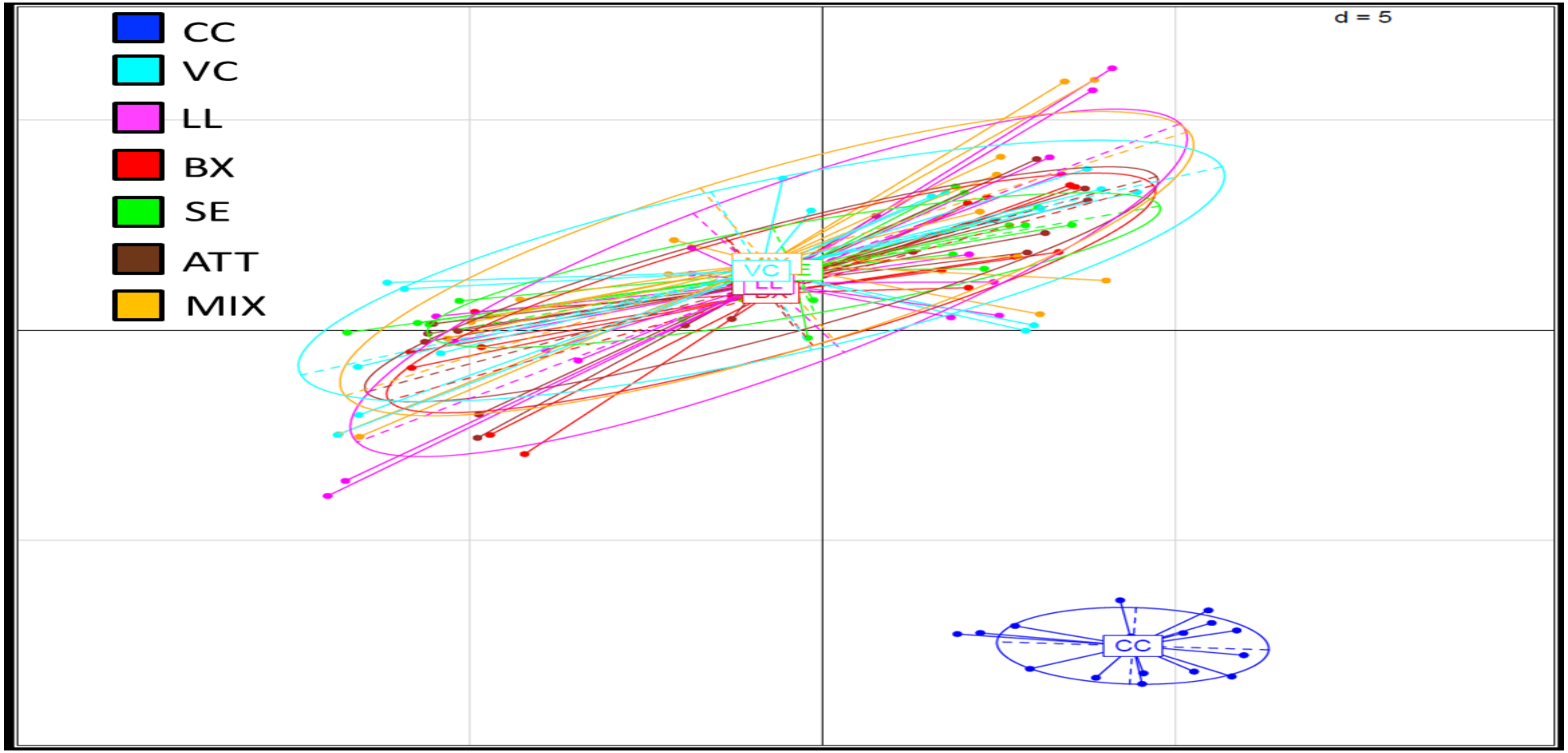

Figure S2. Principal coordinate analysis (PCoA) based on the unweighted UniFrac distance matrix of all 16 S rRNA gene sequences found upon 16S metagenetic analysis performed using the DNA extracted from surface (s) and core (c) level of control cheese (CC), cheese added of attenuated culture of *Lactococcus lactis* subsp. *lactis* CC01 (ATT) and ATT cheeses with the additions, as surface adjunct cultures (2 log CFU/cm^2), of *Leuconostoc*

lactis MC11 (LL), *Vibrio casei* DSM22364 (VC), *Staphylococcus equorum* DSM15097 (SE), *Brochothrix thermosphacta* MC25 (BX) individually or in mix (MIX) during ripening.

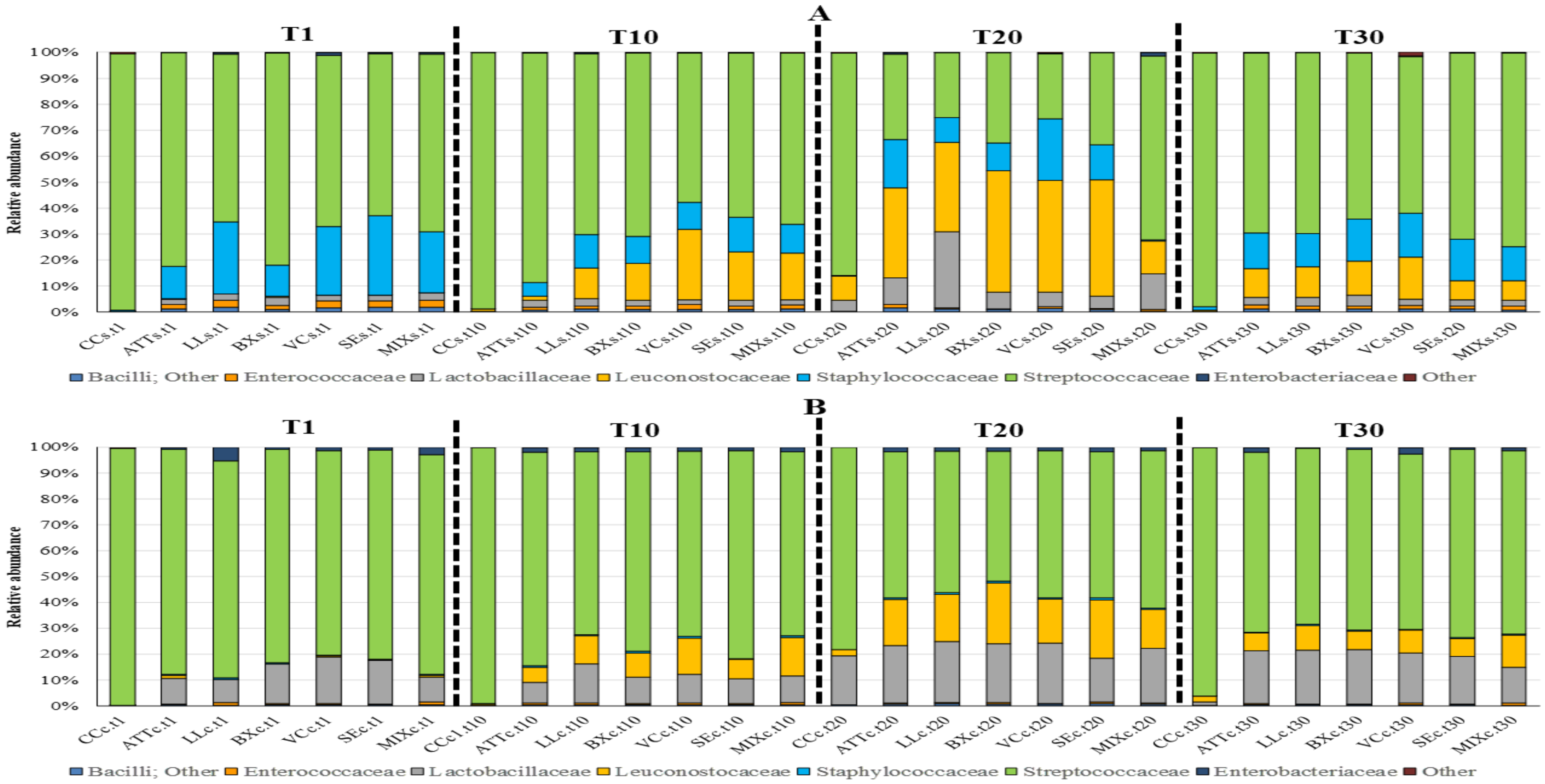

Figure S3. Relative abundance (percent) of total bacteria (family level) found after 1 (T1), 10 (T20), 20 (T20) or 30 (T30) days of ripening in surface (A) and core (B) of control cheese (CC), cheese added of attenuated culture of *Lactococcus lactis* subsp. *lactis* CC01 (ATT) and ATT cheeses with the additions, as surface adjunct cultures (2 log CFU/cm^2), of *Leuconostoc lactis* MC11 (LL), *Vibrio casei* DSM22364 (VC), *Staphylococcus equorum* DSM15097 (SE), *Brochothrix thermosphacta* MC25 (BX) individually or in mix (MIX).

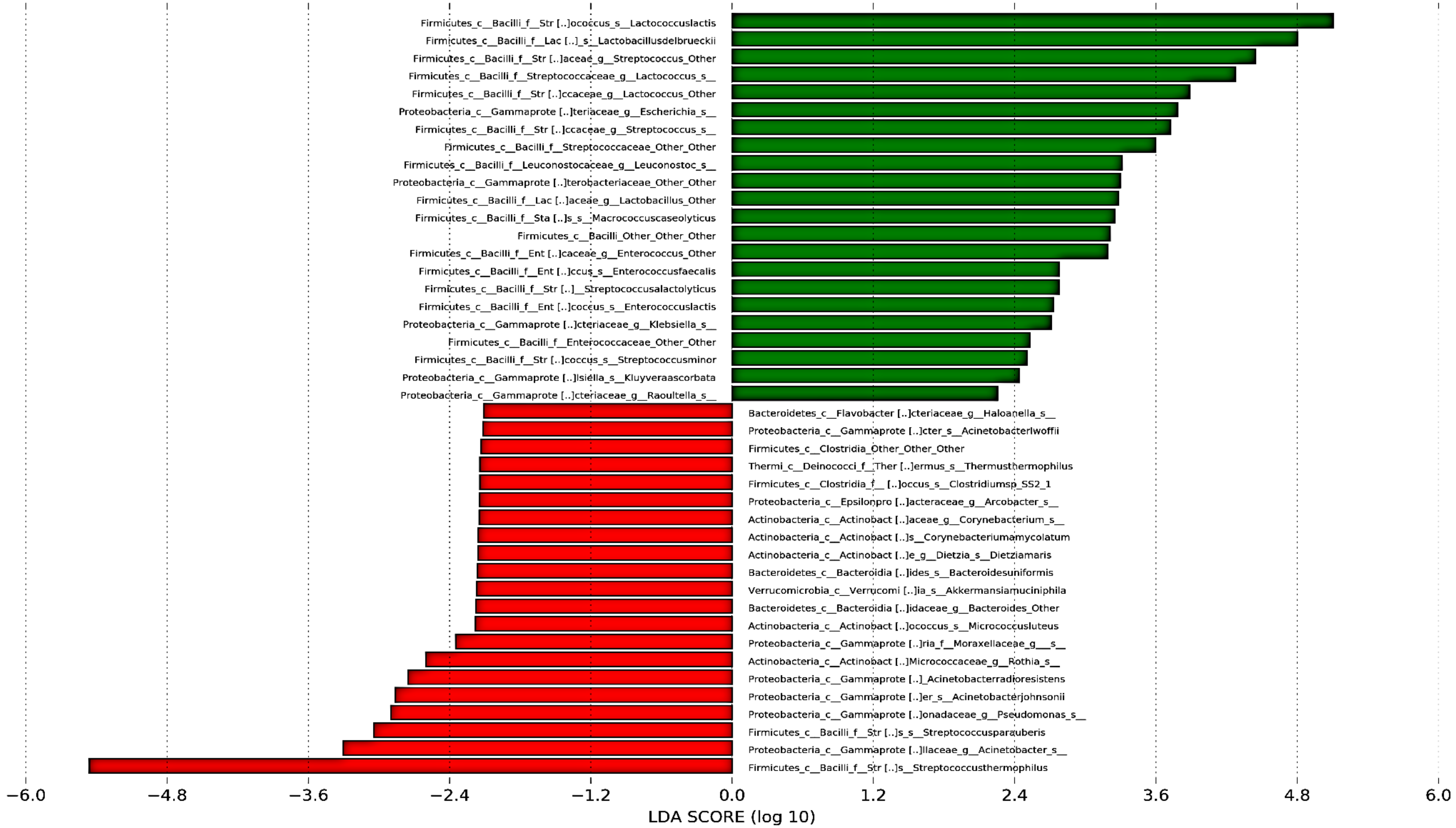

Figure S4. LEfSe analysis of the microbial communities found, after 1 day of ripening, in the rind of control cheese (CC, in red) and the group of cheeses (ATT, LL, VC, SE, BX, MIX, in green) added of attenuated culture of *Lactococcus lactis* subsp. *lactis* CC1.

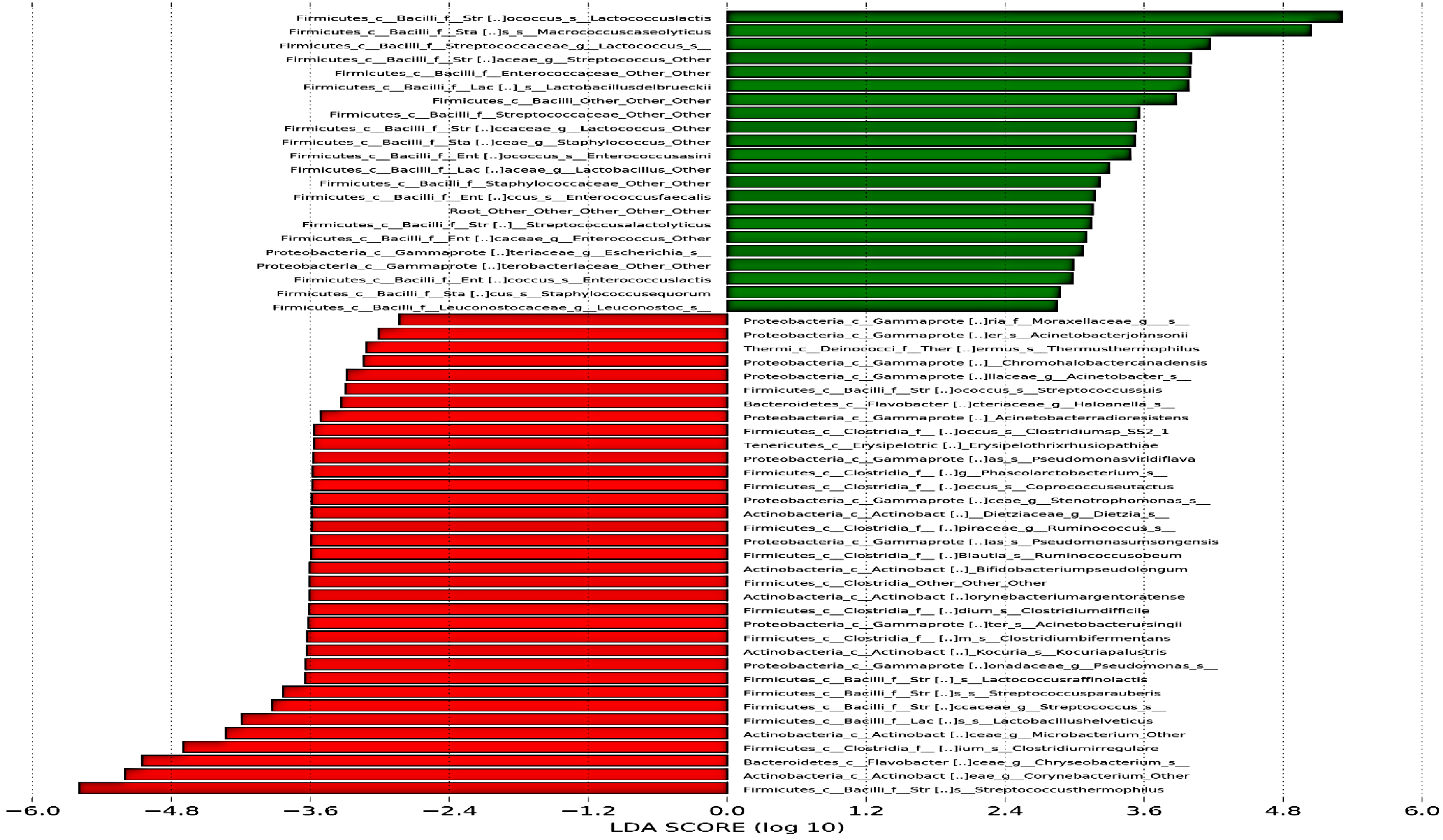

Figure S5. LEfSe analysis of the microbial communities found, after 1 day of ripening, in the core of control cheese (CC, in red) and the group of cheeses (ATT, LL, VC, SE, BX, MIX, in green) added of attenuated culture of *Lactococcus lactis* subsp. *lactis* CC1.

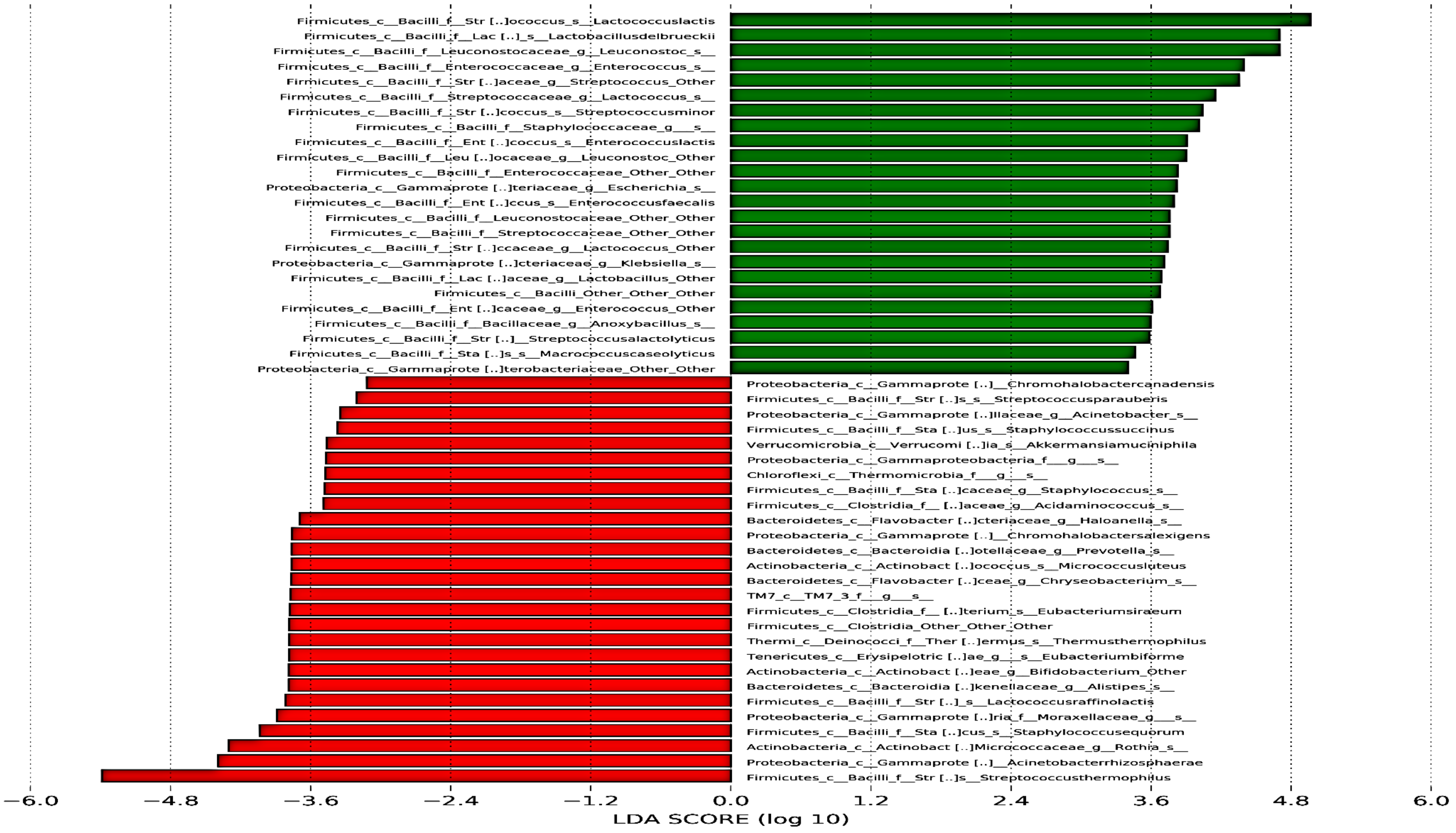

Figure S6. LEfSe analysis of the microbial communities found, after 10 day of ripening, in the rind of control cheese (CC, in red) and the group of cheeses (ATT, LL, VC, SE, BX, MIX, in green) added of attenuated culture of *Lactococcus lactis* subsp. *lactis* CC1.

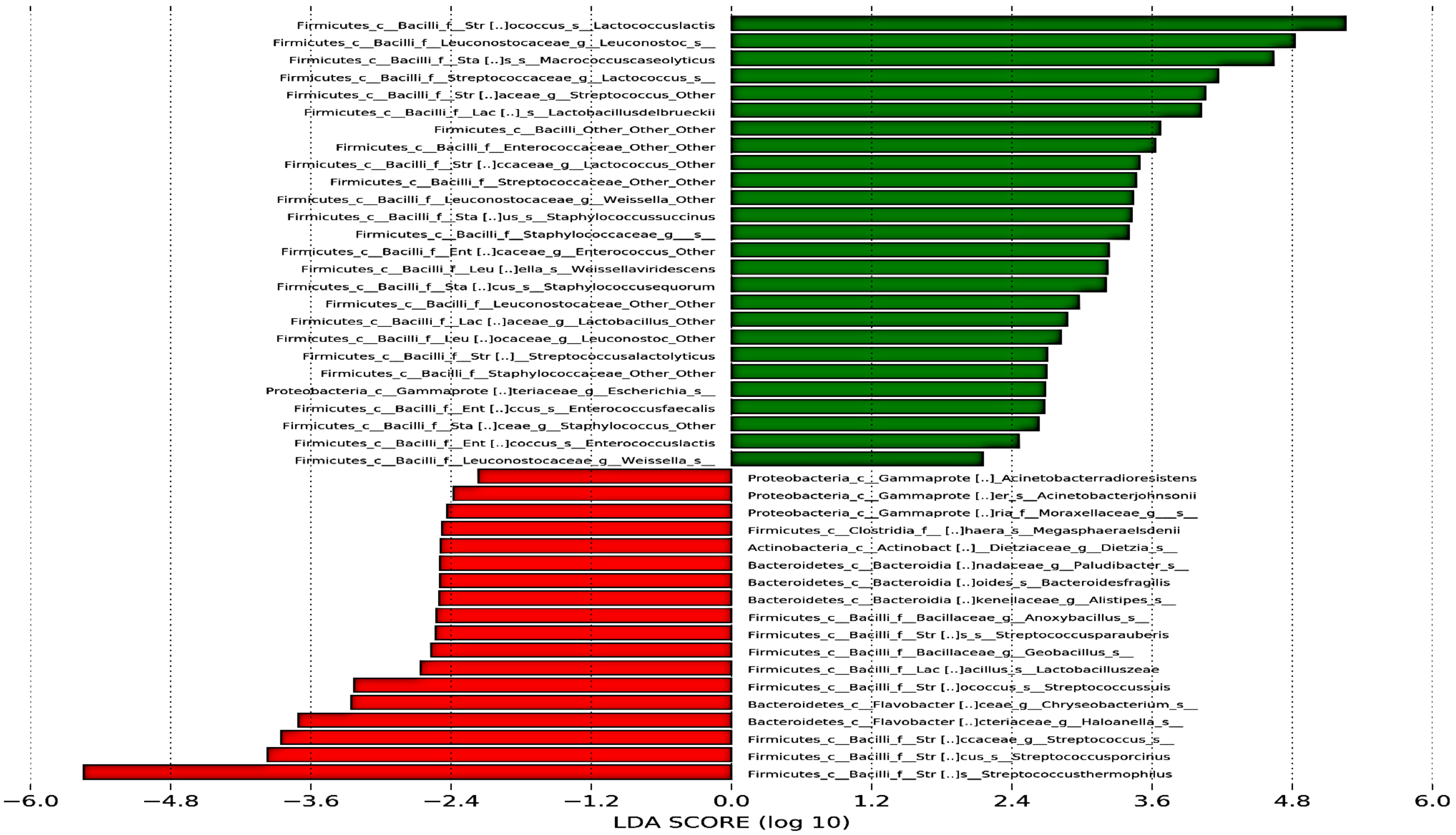

Figure S7. LEfSe analysis of the microbial communities found, after 10 day of ripening, in the core of control cheese (CC, in red) and the group of cheeses (ATT, LL, VC, SE, BX, MIX, in green) added of attenuated culture of *Lactococcus lactis* subsp. *lactis* CC1.

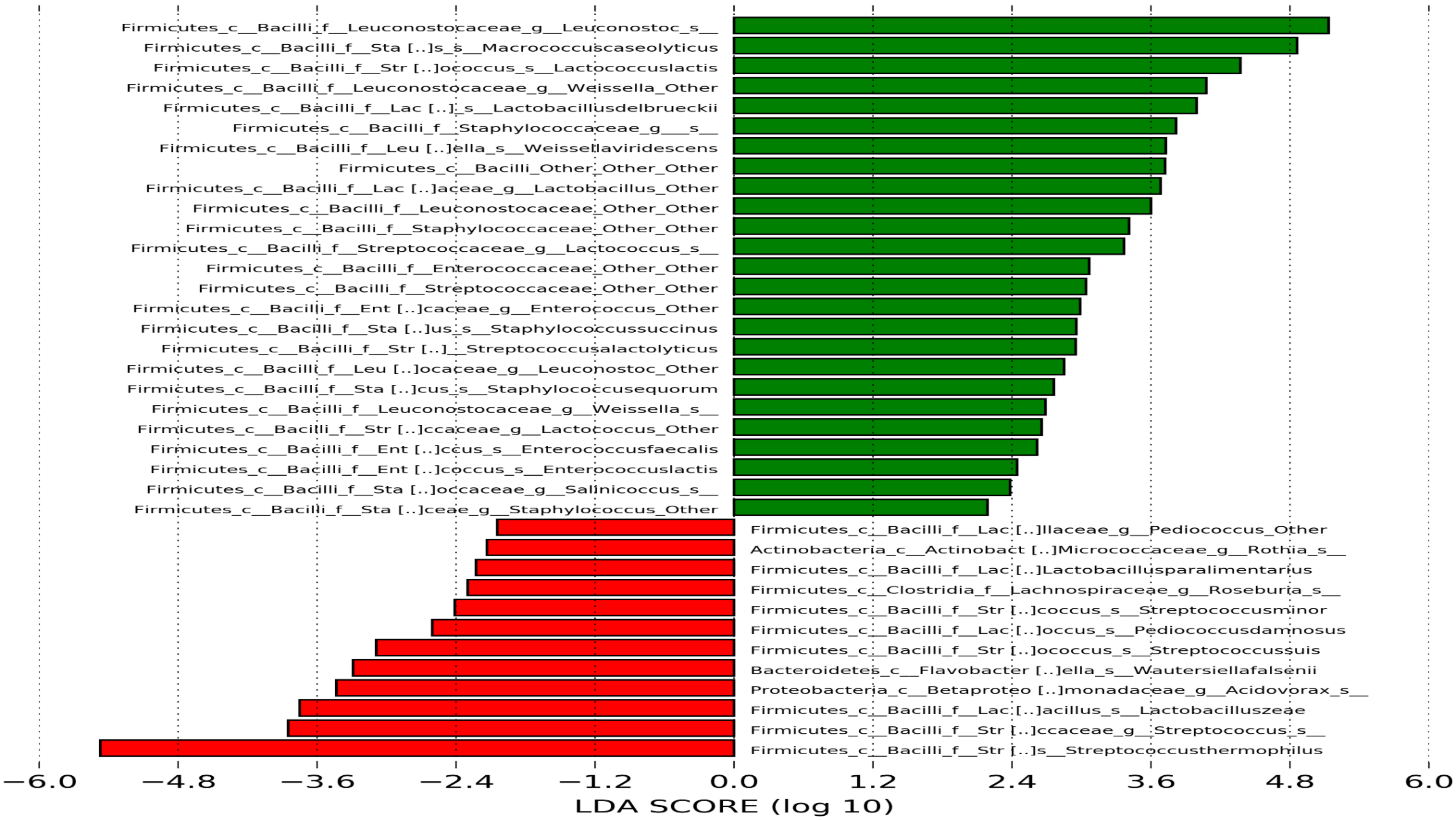

Figure S8. LEfSe analysis of the microbial communities found, after 20 day of ripening, in the rind of control cheese (CC, in red) and the group of cheeses (ATT, LL, VC, SE, BX, MIX, in green) added of attenuated culture of *Lactococcus lactis* subsp. *lactis* CC1.

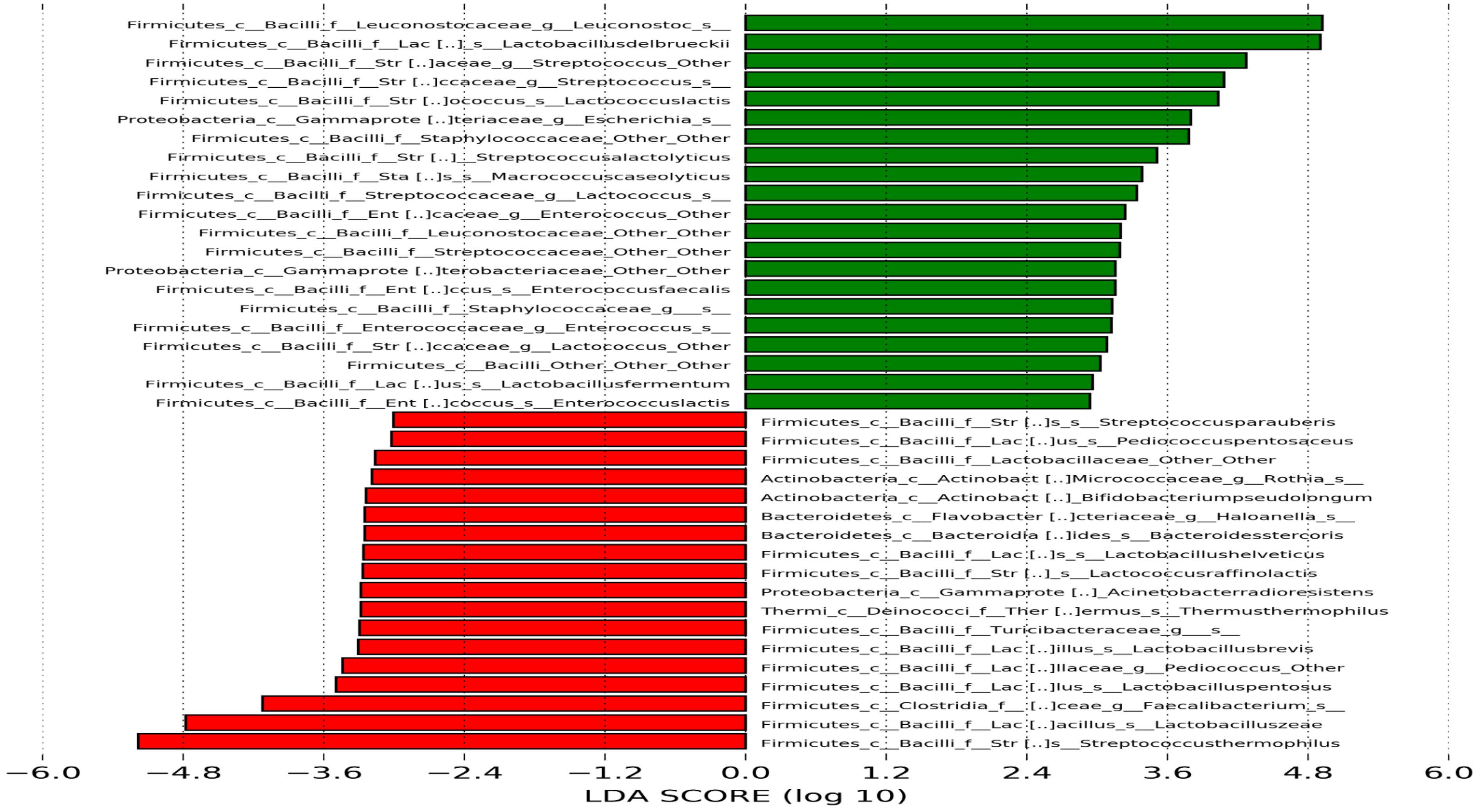

Figure S9. LEfSe analysis of the microbial communities found, after 20 day of ripening, in the core of control cheese (CC, in red) and the group of cheeses (ATT, LL, VC, SE, BX, MIX, in green) added of attenuated culture of *Lactococcus lactis* subsp. *lactis* CC1.

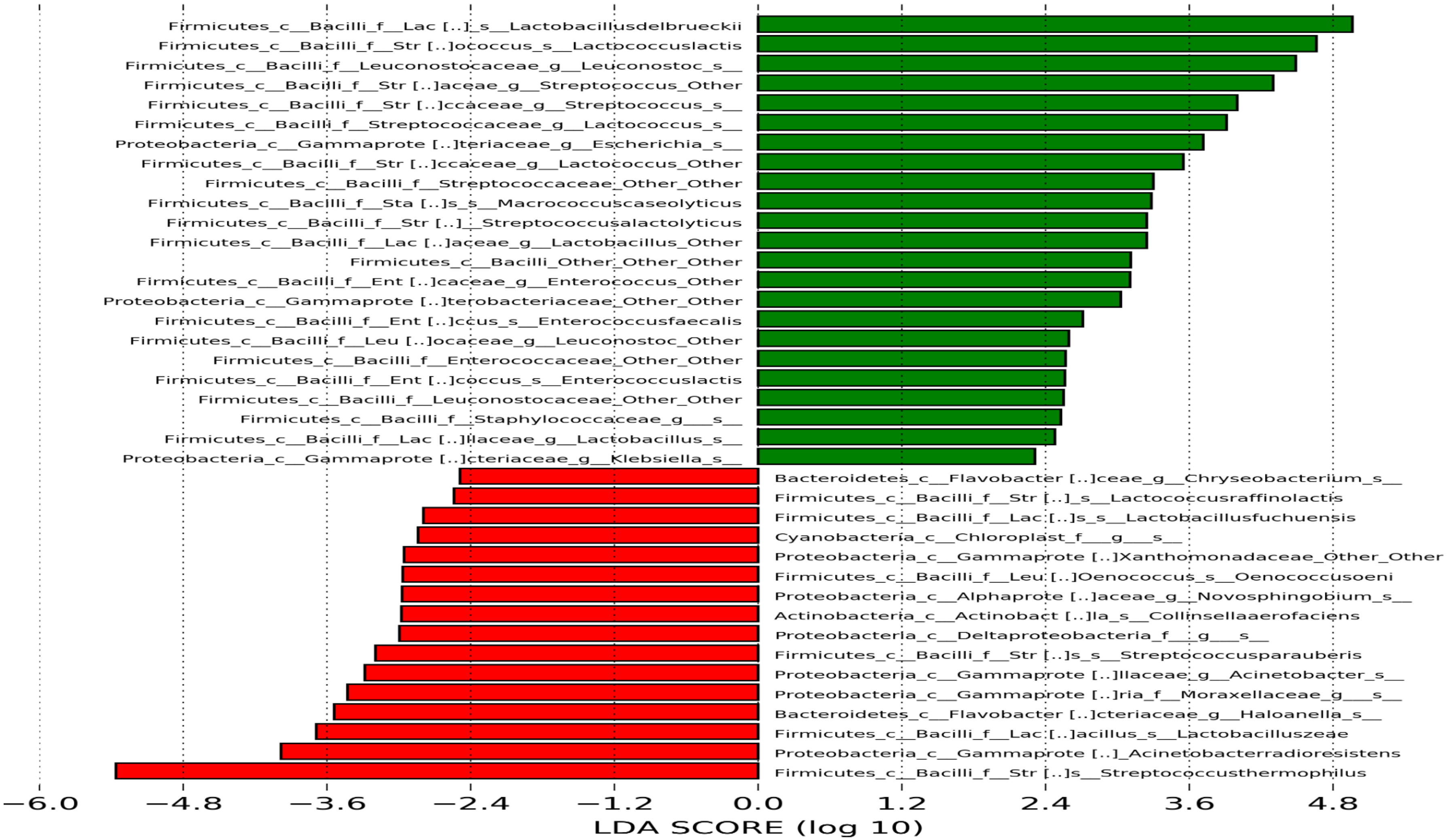

Figure S10. LEfSe analysis of the microbial communities found, after 30 day of ripening, in the rind of control cheese (CC, in red) and the group of cheeses (ATT, LL, VC, SE, BX, MIX, in green) added of attenuated culture of *Lactococcus lactis* subsp. *lactis* CC1.

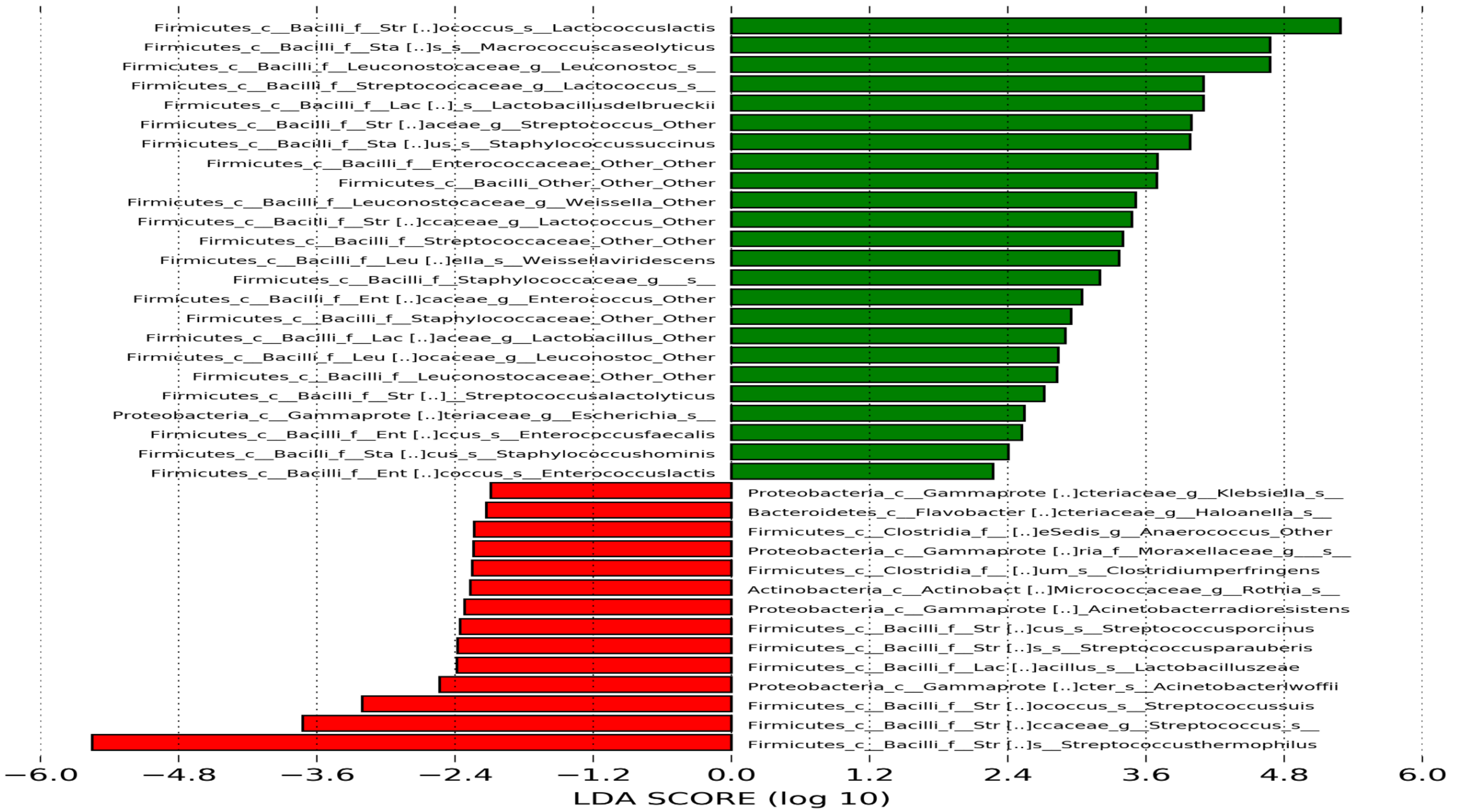

Figure S11. LEfSe analysis of the microbial communities found, after 30 day of ripening, in the core of control cheese (CC, in red) and the group of cheeses (ATT, LL, VC, SE, BX, MIX, in green) added of attenuated culture of *Lactococcus lactis* subsp. *lactis* CC1.

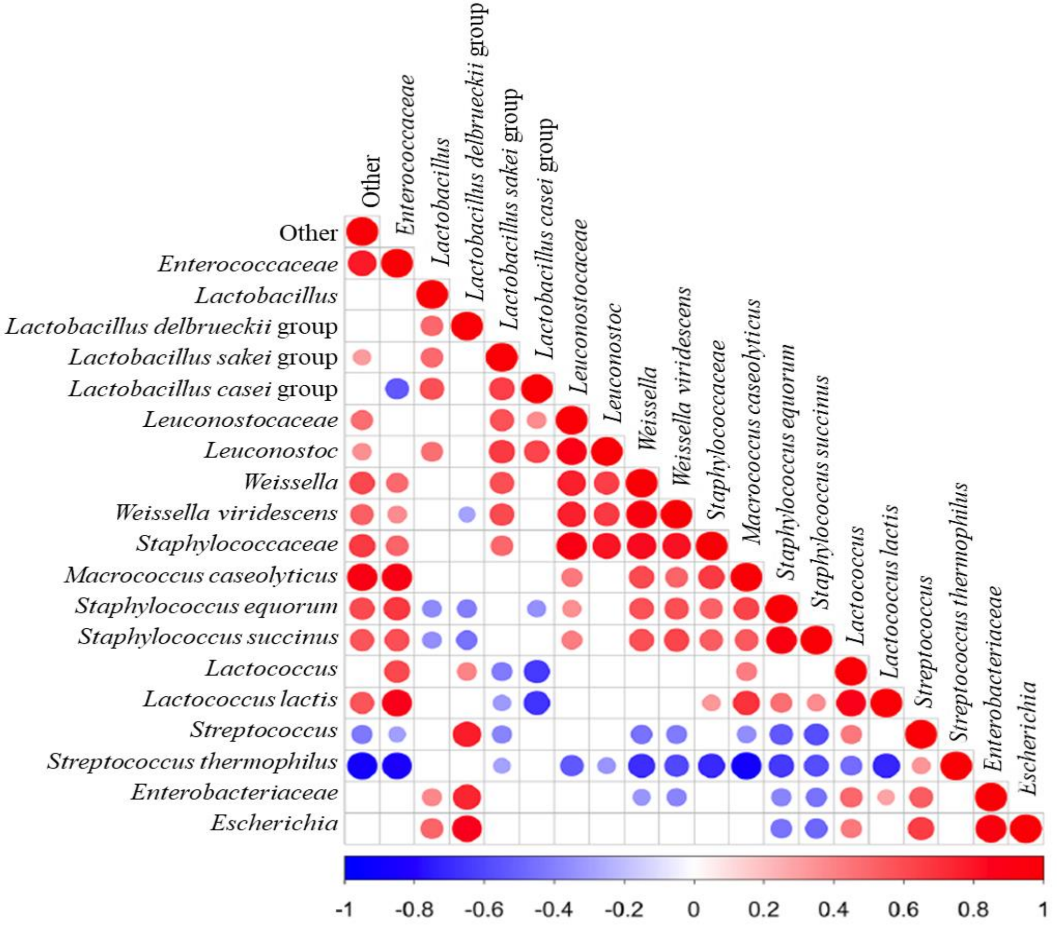

Figure S12. Significant co-occurrence and co-exclusion relationships between bacteria found in cheeses during ripening, through 16S metagenetic analysis. Spearman's rank correlation matrix was used. Strong correlations are indicated by large circles, whereas weak correlations are indicated by small circles. The colors of the scale bar denote the nature of the correlation, with 1 indicating a perfectly positive correlation (dark red) and -1 indicating a perfectly negative correlation (dark blue) between two bacterial OTUs. Only significant correlations (FDR < 0.05) are shown.

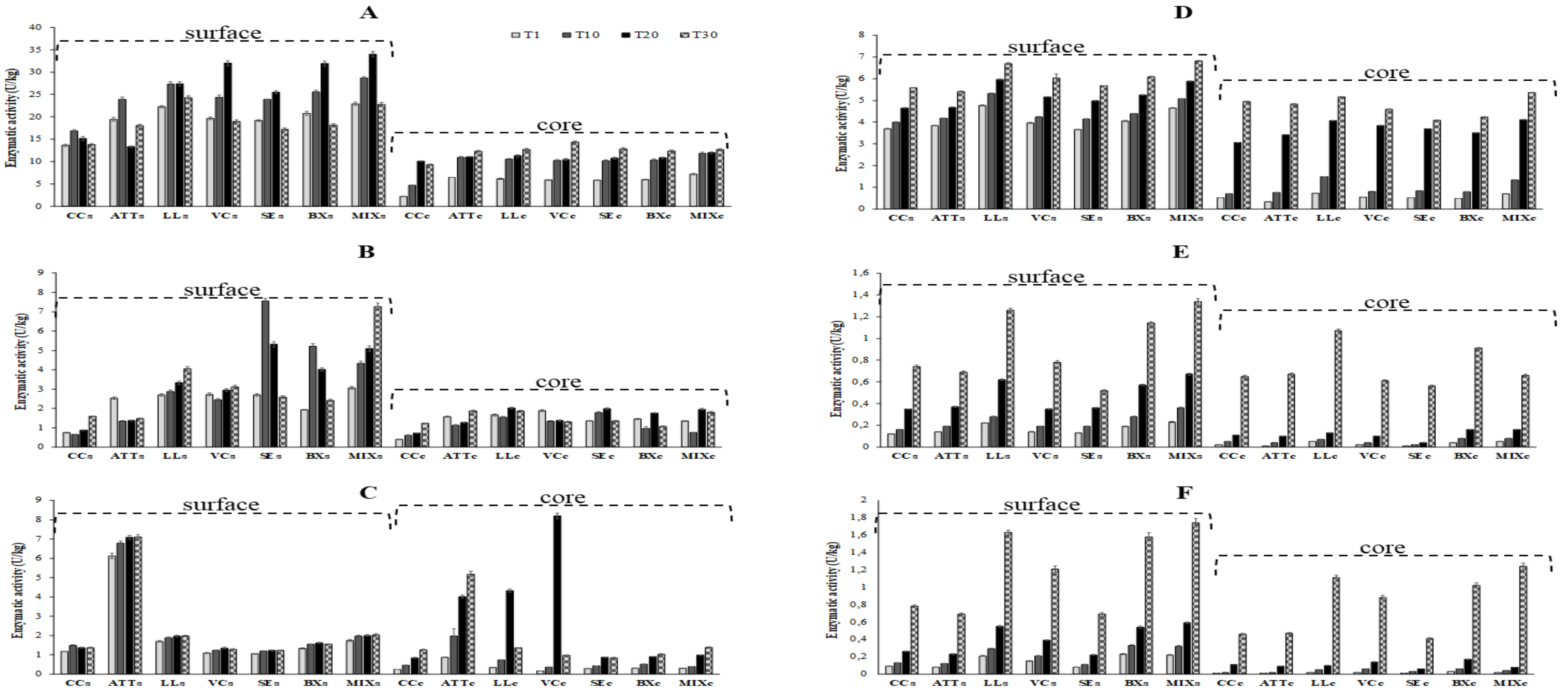

Figure S13. Enzymatic activities found at surface (s) and core (c) level of control cheese (CC), cheese added of attenuated culture of *Lactococcus lactis* subsp. *lactis* CC01 (ATT) and ATT cheeses with the additions, as surface adjunct cultures (2 log CFU/cm^2), of *Leuconostoc lactis* MC11 (LL), *Vibrio casei* DSM22364, (VC), *Staphylococcus equorum* DSM15097 (SE), *Brochothrix thermosphacta* MC25 (BX) individually or in mix (MIX), after 1 (T1), 10 (T10), 20 (T20) or 30 (T30) days of ripening. Panels show activities of aminopeptidase type N (A), proline iminopeptidase (B),

endopeptidase type O (C), glutamate dehydrogenase (D), cystathionine-γ-lyase (E), and esterase (F). Values represent the average (± SD) of analysis performed on three samples coming from as many cheese-making experiments.

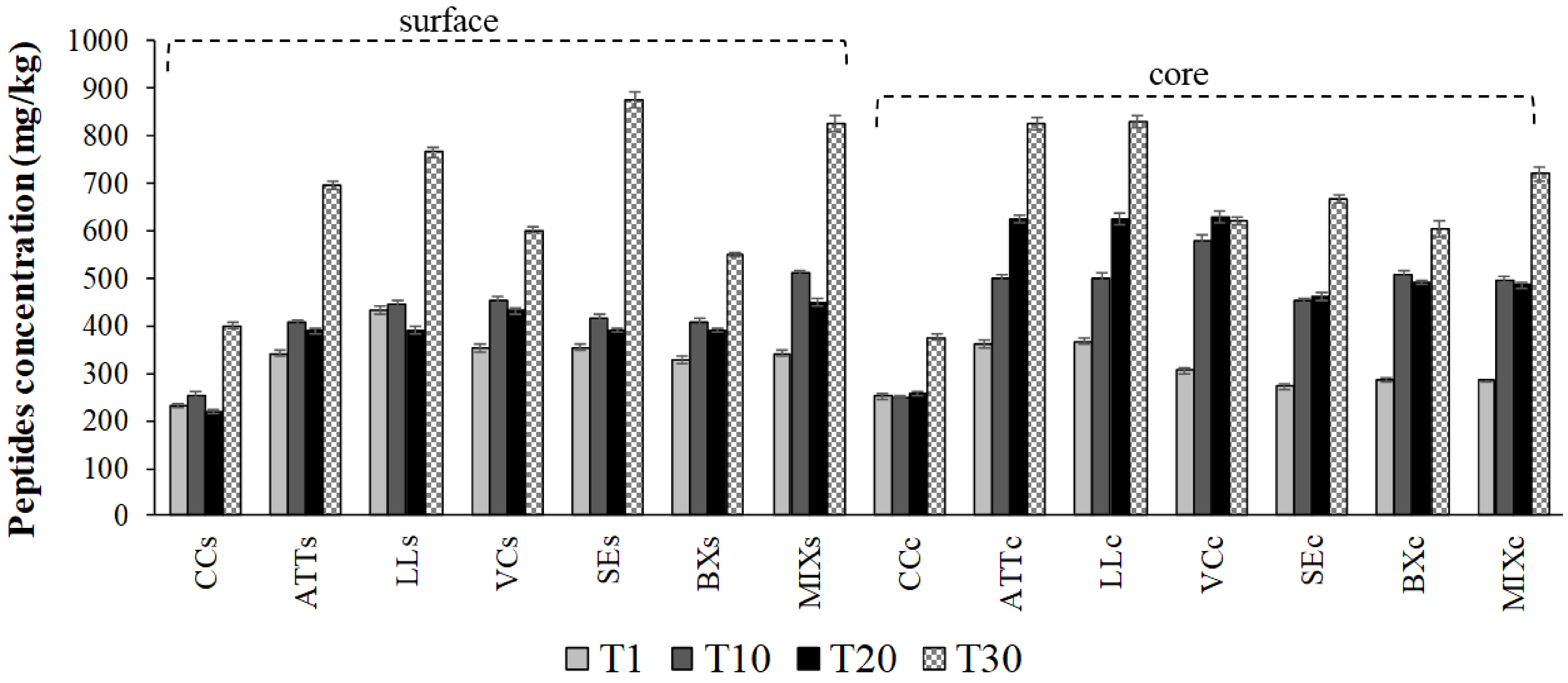

Figure S14. Peptides concentration found at surface (s) and core (c) level of control cheese (CC), cheese added of attenuated culture of *Lactococcus lactis* subsp. *lactis* CC01 (ATT) and ATT cheeses with the additions, as surface adjunct cultures (2 log CFU/cm^2), of *Leuconostoc lactis* MC11 (LL), *Vibrio casei* DSM22364 (VC), *Staphylococcus equorum* DSM15097 (SE), *Brochothrix thermosphacta* MC25 (BX) individually or in mix (MIX), after 1 (T1), 10 (T10), 20 (T20) or 30 (T30) days of ripening. Values represent the average (± SD) of analysis performed on three samples coming from as many from as many cheese-making experiments.

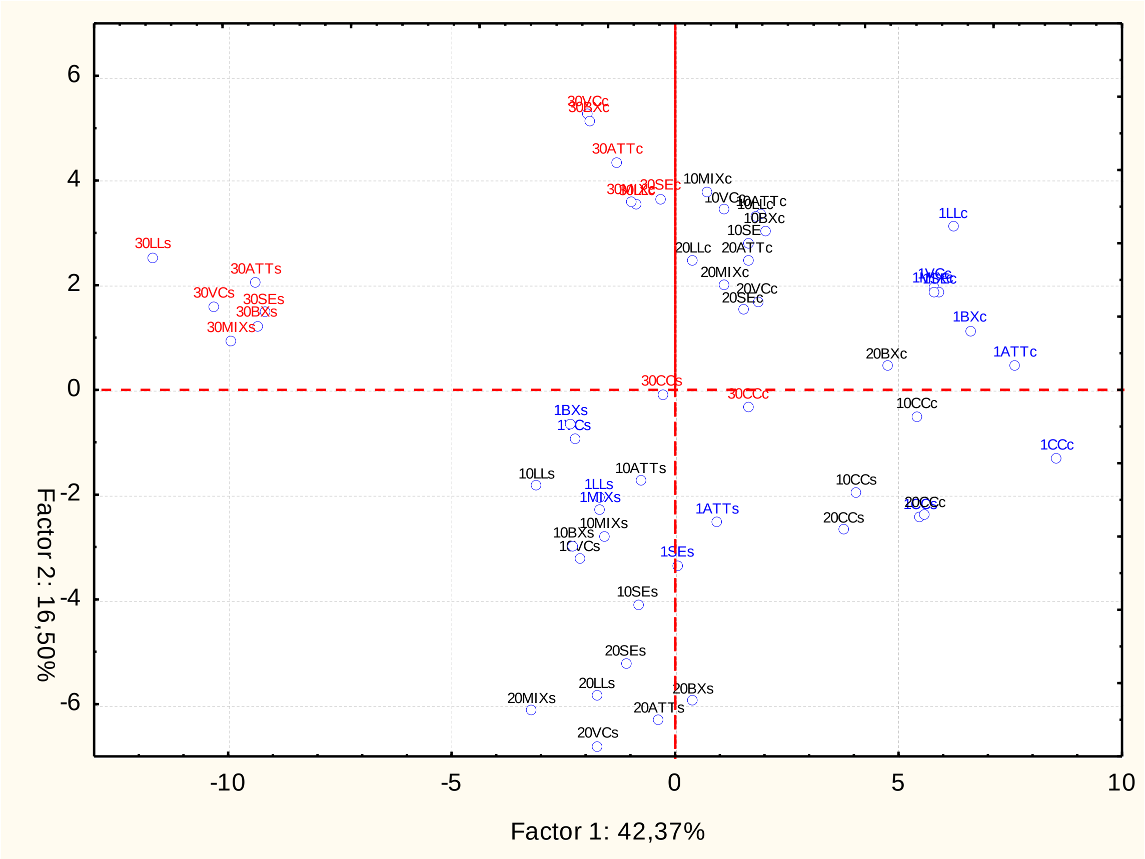

Figure S15A. Score plot of first and second principal components (PC) after PC analysis based on cultivable and total bacteria, peptides, enzymatic activities and individual and total free amino acids that mainly ($P < 0.05$) differentiated at surface (s) and core (c) level the control cheese (CC), cheese added of attenuated culture of *Lactococcus lactis* subsp. *lactis* CC01 (ATT) and ATT cheeses with the additions, as surface adjunct cultures (2 log CFU/cm^2), of *Leuconostoc lactis* MC11 (LL)*, Vibrio casei* DSM22364 (VC)*, Staphylococcus equorum* DSM15097 (SE)*, Brochothrix thermosphacta* MC25 (BX) individually or in mix (MIX), after 1, 10, 20 or 30 days of ripening.

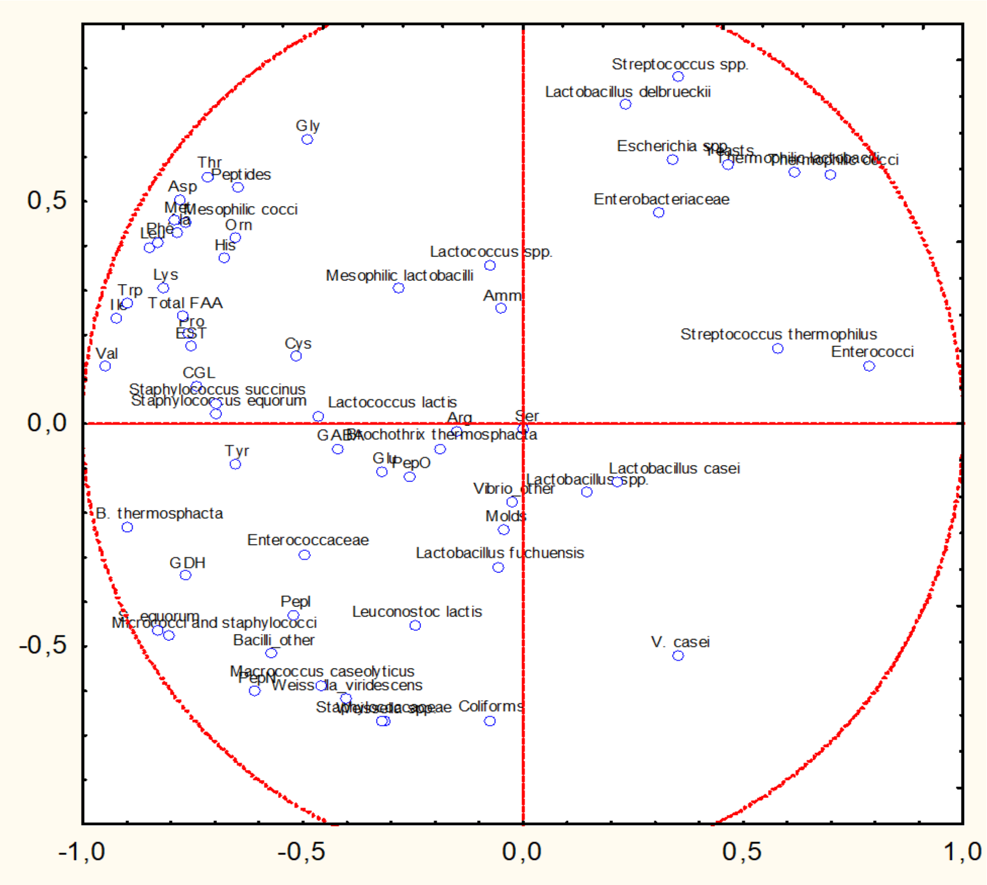

Figure S15B. Loading plot of first and second principal components (PC) after PC analysis based on cultivable and total bacteria, peptides, enzymatic activities and individual and total free amino acids that mainly ($P < 0.05$) differentiated at surface (s) and core (c) level the control cheese (CC), cheese added of attenuated culture of *Lactococcus lactis* subsp. *lactis* CC01 (ATT) and ATT cheeses with the additions, as surface adjunct cultures (2 log CFU/cm^2), of *Leuconostoc lactis* MC11 (LL), *Vibrio casei* DSM22364 (VC), *Staphylococcus equorum* DSM15097 (SE), *Brochothrix thermosphacta* MC25 (BX) individually or in mix (MIX), after 1, 10, 20 or 30 days of ripening.

MATERIALS AND METHODS

Kinetics of growth. Cells of *Leuc. lactis*, *V. casei*, *B. thermosphacta* and *St. equorum* grown on the respective broth media for 24 h at 30°C were harvested by centrifugation, washed with sterile phosphate buffer 50 mM pH 7.0, and re-suspended at the cell density of ca. 7.0 log CFU/mL. Each bacterial suspension was singly used to inoculate the CB medium (71) at a final cell density of 2.0 log CFU/ml. The incubation was at room temperature (25 °C) for the first 12 h and further incubated at 9 °C for ten days. At intervals of 24 h, cell density was estimated by plating on the respective agar media.

Enzyme activities of attenuated and surface adjunct cultures. The enzymatic activities were determined as previously described by De Angelis et al. (72). In detail, *Lc. lactis*, *Leuc. lactis*, *V. casei*, *B. thermosphacta* and *St. equorum* were singly inoculated in CB medium at an initial cell density of 7.0 log CFU/ml and incubated at 30 °C for 24 h. After incubation, cells were collected by centrifugation (10,000 × g, 10 min at 4°C), washed in sterile phosphate buffer 50 mM pH 7.0, and used for enzymatic assays at a final cell density of 8.5±0.3 log CFU/ml. Aminopeptidase (EC 3.4.11.11, pepN) type N activity on Leu-*p*-nitroanilide (Leu-*p*NA), proline iminopeptidase (EC 3.4.11.9, pepI) activity on Pro-*p*-nitroanilide (Pro-*p*NA), and endopeptidase type O (EC 3.4.23, pepO) activity on Z-Gly-Pro-NH-trifluoromethylcoumarin (Z-Gly-Pro-NA) were determined as described by Gobbetti et al. (62). An arbitrary unit of enzymatic activity was defined as the amount of enzymethat caused an increase in absorbance at 410 nm of 1 (pepN) and 0.1 (pepI and pepO) per minute at 37°C and pH 7.0. The assessment of glutamate dehydrogenase (EC 1.4.1.2, GDH) activity was on glutamate by measuring the glutamate-dependent reduction of NADP or NAD at 340 nm as described by De Angelis et al. (63). An arbitrary unit of enzymatic activity was defined as the amount of enzyme that gave an increase of absorbance of 0.1 per minute at 37°C and pH 7.0. Cystathionine gamma-lyase (EC 4.4.1.1, CGL) activity was determined by measuring the amount of ketoacids, ammonia, and free thiols released from cystathionine, as described by De Angelis et al. (73). An

arbitrary unit of enzymatic activity was defined as the amount of enzyme that caused an increase of absorbance (412 nm) of 1 per minute at 37°C and pH 7.0. The esterase activity was determined using α-naphthyl butyrate as the substrate (74). A unit of esterase activity was defined as the amount of enzyme that released 1 μmol of α-naphthol per minute at 37°C and pH 7.0.

Table S3. Definition of the descriptors used during the sensory analysis of Caciotta cheese (adapted from (66) and (67).

Descriptor	Definition
Uniformity of crust color	Overall uniformity in rind color: presence of either shades or tones reduces uniformity
Uniformity of core color	Overall uniformity in core color: presence of either shades or tones reduces uniformity
Internal structure	Overall uniformity in structure: presence of either fissures or holes reduces uniformity
Odor intensity	Odor arising from butter at room temperature
Butter taste	Flavor arising from butter at room temperature
Rancid smell	Odor arising from rancid butter at room temperature
Spicy smell	Odor arising from pepper at room temperature
Aroma intensity	Organoleptic attribute perceptible by the olfactory organ via the back of the nose when tasting
Taste intensity	Taste recalling milk and butter
Moisture	Moisture released by the product in the mouth during early mastication (dry: saliva is absorbed by the product; humid: liquids are released during mastication)
Acid	Fundamental taste associated with citric acid
Bitter	Fundamental taste associated with quinine
Salty	Fundamental taste associated with sodium chloride
Sweet	Fundamental taste associated with sucrose

REFERENCES

21. Calasso M, Ercolini D, Mancini L, Stellato G, Minervini F, Di Cagno R, De Angelis M, Gobbetti M. 2016. Relationships among house, rind and core microbiotas during manufacture of traditional Italian cheeses at the same dairy plant. Food Microbiol 54:115–126.

62. Gobbetti M, Folkertsma B, Fox PF, Corsetti A, Smacchi E, De Angelis M, Rossi J, Kilcawley K, Cortini M. 1999. Microbiology and biochemistry of Fossa (pit) cheese. Int Dairy J 9:763–773.

63. De Angelis M, Calasso M, Di Cagno R, Minervini F, Gobbetti M. 2010. NADP-glutamate dehydrogenase activity in non-starter lactic acid bacteria: Effects of temperature, pH and NaCl on enzyme activity and expression. J Appl Microbiol 109:1763–1774.

66. Niro S, Fratianni A, Tremonte P, Sorrentino E, Tipaldi L. 2014. Innovative Caciocavallo cheeses made from a mixture of cow milk with ewe or goat milk. J Dairy Sci 97:1296–1304.

67. Albenzio M, Santillo A, Caroprese M, Braghieri A, Sevi A, Napolitano F. 2013. Composition and sensory profiling of probiotic Scamorza ewe milk cheese. J Dairy Sci 96 :2792–2800.

69. Bleicher A, Neuhaus K, Scherer S. 2010. *Vibrio casei* sp. nov., isolated from the surfaces of two French red smear soft cheeses. Int J Syst Evol Microbiol 60:1745–1749.

70. Place RB, Hiestand D, Gallmann HR, Teuber M. 2003. *Staphylococcus equorum* subsp. *linens*, subsp. nov., a starter culture component for surface ripened semi-hard cheeses. Syst Appl Microbiol 26:30–37.

71. Neviani E, De Dea Lindner J, Bernini V, Gatti M. 2009. Recovery and differentiation of long ripened cheese microflora through a new cheese-based cultural medium. Food Microbiol 26:240–245.

72. De Angelis M, Di Cagno R, Gallo G, Curci M, Siragusa S, Crecchio C, Parente E, Gobbetti M. 2007. Molecular and functional characterization of *Lactobacillus sanfranciscensis* strains isolated from sourdoughs. Int J Food Microbiol 114:69–82.

73. De Angelis M, Curtin AC, McSweeney PLH, Faccia M, Gobbetti M. 2002. *Lactobacillus reuteri* DSM 20016: Purification and characterization of a cystathionine γ–lyase and use as adjunct starter in cheesemaking. J Dairy Res 69:255–267.75.

74. De Angelis M, Gobbetti M, Corsetti A. 1999. Esterase and lipase activities of *Lactobacillus sanfranciscensis* strains used in sourdough fermentation. Ital J Food Sci 11:167–172.
